# The Hippo signalling pathway in bone homeostasis: Under the regulation of mechanics and aging

**DOI:** 10.1111/cpr.13652

**Published:** 2024-05-03

**Authors:** Zhengda Li, Junqing Lin, Jing Wu, Jinlong Suo, Zuoyun Wang

**Affiliations:** ^1^ Department of Human Anatomy and Histoembryology, School of Basic Medical Sciences and Shanghai Jing'an District Central Hospital Fudan University Shanghai China; ^2^ Institute of Microsurgery on Extremities, and Department of Orthopedic Surgery Shanghai Sixth People's Hospital Affiliated to Shanghai Jiao Tong University School of Medicine Shanghai Shanghai China

## Abstract

The Hippo signalling pathway is a conserved kinase cascade that orchestrates diverse cellular processes, such as proliferation, apoptosis, lineage commitment and stemness. With the onset of society ages, research on skeletal aging‐mechanics‐bone homeostasis has exploded. In recent years, aging and mechanical force in the skeletal system have gained groundbreaking research progress. Under the regulation of mechanics and aging, the Hippo signalling pathway has a crucial role in the development and homeostasis of bone. We synthesize the current knowledge on the role of the Hippo signalling pathway, particularly its downstream effectors yes‐associated protein (YAP) and transcriptional co‐activator with PDZ‐binding motif (TAZ), in bone homeostasis. We discuss the regulation of the lineage specification and function of different skeletal cell types by the Hippo signalling pathway. The interactions of the Hippo signalling pathway with other pathways, such as Wnt, transforming growth factor beta and nuclear factor kappa‐B, are also mentioned because of their importance for modulating bone homeostasis. Furthermore, YAP/TAZ have been extensively studied as mechanotransducers. Due to space limitations, we focus on reviewing how mechanical forces and aging influence cell fate, communications and homeostasis through a dysregulated Hippo signalling pathway.

## Graphical abstract

The Hippo signalling pathway modulates the lineage specification and function of different skeletal cell types. Mechanical forces and aging influence bone homeostasis through a dysregulated Hippo signalling pathway.

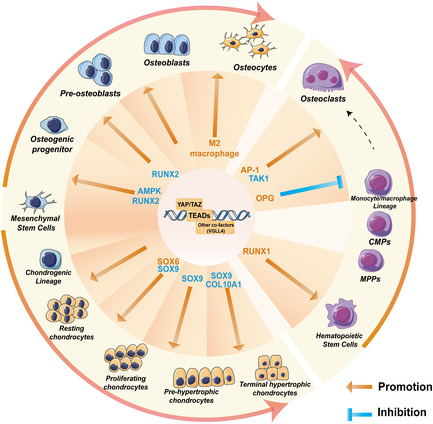

## 1 INTRODUCTION

Bone homeostasis is a dynamic process that encompasses bone formation, maintenance and resorption, all of which are crucial for preserving the strength and integrity of the skeleton. Disruptions in bone homeostasis can lead to a variety of bone‐related diseases. The maintenance of bone homeostasis is a complex and finely tuned process, influenced by multiple factors such as intercellular communication,^1^ neural regulation,^2^ immune responses within the bone microenvironment,^3^ mechanical forces and aging factors. Consequently, researching the factors that affect bone homeostasis is vital for addressing bone‐related diseases.

The Hippo signalling pathway is a kinase cascade that modulates key biological functions, particularly bone homeostasis.^4^ Through its main transcriptional co‐factors, Yes‐associated protein (YAP) and transcriptional co‐activator with PDZ‐binding motif (TAZ), the Hippo pathway influences the lineage commitment and regulation of various bone‐related cell populations. From the perspective of stem cells, we review the various bone‐related cell populations and highlight the Hippo signalling pathway in their lineage commitment and proliferation. We also review the crosstalk between the Hippo signalling pathway and other signalling pathways, such as Wingless/Integrated (Wnt), transforming growth factor beta (TGF‐β) and nuclear factor kappa‐B (NF‐κB), that regulate the function of each bone cell type.

We explore how mechanical forces and aging, as two major factors that influence Hippo signalling, affect bone homeostasis. It is highlighted how alterations in Hippo pathway activity due to these factors can disrupt the equilibrium of bone maintenance. The response of the Hippo signalling pathway to mechanical cues, oxidative stress, oncogene activation, RNA dysregulation and other aging‐related factors is scrutinized.^5^, ^6^ Such modulations are linked to the development of aging‐related conditions, including osteoarthritis and osteoporosis. Furthermore, the interplay between aging, mechanical cues and the Hippo signalling pathway in bone homeostasis is examined. In this review, we examine the intricate role of the Hippo signalling pathway in bone homeostasis, highlighting its interaction with various bone‐related cell populations and other regulatory pathways, and how factors like mechanical forces and aging factors can disrupt this homeostasis, potentially leading to bone diseases.

## 2 BONE‐RELATED CELL POPULATIONS

### 2.1 Mesenchymal stem cells and skeletal stem cells

Mesenchymal stem cells (MSCs) as pluripotent adult stem cells can be found in different tissues, including bone marrow, adipose, synovium and blood.^7^, ^8^ The International Society for Cellular Therapy identifies MSCs lineages by positive markers like Cluster of Differentiation 90 (*CD90*), *CD73* and *CD105*.^9^ Divided from bone marrow and identified by Friedenstein in 1976, bone marrow MSCs (BMSCs) are the first divided MSCs and the most thoroughly studied MSCs. Synovium MSCs are synovium‐derived *Gdf5*‐lineage cells.^7^ They express MSCs surface markers and proliferate after peri‐synovial cartilage injury to repair.^7^ Since synovium MSCs are easy to isolate and have strong cartilage differentiation potential, they are considered key to the treatment of osteoarthritis and rheumatoid arthritis.^10^ Scaffolds containing synovium MSCs, exosomes of miR‐140‐5p overexpressed synovium MSCs and small molecule drugs such as SHP099 all promote chondrocyte proliferation and migration to the injured site, demonstrating hope for curing joint cartilage injury, even osteoarthritis and rheumatoid arthritis.^11^, ^12^

SSCs are skeletal lineage stem cells, differentiating into osteoblasts and chondrocytes but not adipocytes.^13^, ^14^ Although there are many functional and differentiation similarities with MSCs of skeletal lineage, it is believed that SSCs exist at the vertex of their differentiation level.^15^ Human SSCs are defined with cell surface markers like Podoplanin (PDPN)^+^ CD164^+^ CD73^+^ CD146^−^.^14^ SSCs are more focused on the differentiation of the skeletal lineages, therefore they are more susceptible to research on mechanical forces, aging and diseases compared with MSCs.^16^ Periosteal stem cells (PSCs) are one kind of periosteal‐divided SSCs, differentiated from MSCs by expressing cathepsin K (Ctsk) and differentiated from osteoclasts which also express Ctsk by periosteal localization.^17^, ^18^ PSCs are CD45^−^ lymphocyte antigen 76 (Ly76)^−^ CD31^−^ CD90^−^ 6C3^−^ CD49f^dim^ CD51^dim^ CD200^+^ CD105^−^ Ctsk^+^ cells in the periosteum.^18^ Derived from the periosteum, PSCs directly differentiate into osteoblasts through the intramembrane ossification.^18^ However, the lineage commitment of PSCs is not immutable, as PSCs could also differentiate into chondrocytes during fracture healing.^19^, ^20^

#### 2.1.1 *Runx2* commits the osteoblast lineage of MSCs

MSCs differentiate into osteoblast lineages through Runt‐related transcription factor 2 (*Runx2*), a critical skeletal transcription factor. *Runx2* regulates Indian hedgehog (Ihh), fibroblast growth factor (Fgf) and Wnt pathways for osteoblast commitment and maturation.^21^, ^22^ Bone morphogenetic protein (BMP) family also contributes to the osteoblast‐lineage commitment of MSCs.^23^ These osteoblast‐lineage MSCs form osteoblasts through the pre‐osteoblasts stage after lineage commitment and express alkaline phosphatase, biomineralization associated (ALPL), osterix (Osx) and osteoprotegerin (OPG).^24^ These pre‐osteoblasts stage cells called osteogenic progenitors are marked with Leptin Receptor (Lepr), Osteolectin (Oln) and GLI‐Kruppel family member GLI1 (Gli1).^25^, ^26^ Some osteoblasts expressing dentin matrix protein 1 (DMP1) and matrix metalloproteinases (MMPs) differentiate into osteocytes by terminal differentiation.^27^ Osteoblasts are intermittently active to differentiate into osteocytes and can revert to bone lining cells when quiescent.^27^

#### 2.1.2 *Sox9* commits the chondrocytes lineage of MSCs

SRY‐box transcription factor 9 (*Sox9*) regulates the multi‐process of chondrocyte development by enhancing the expression of chondrocyte‐related genes.^28^, ^29^ Microtubule‐associated serine/threonine kinase family member 4 (*Mast4*) suppression activates *Sox9* for chondrocyte‐lineage commitment of MSCs.^30^ This lineage commitment process, called condensation, upregulates chondrocyte‐lineage markers like collagen type II alpha 1 chain (COL2A1), Aggrecan (ACAN), Sox9 and collagen type IX alpha 1 chain (COL9A1), while downregulates MSCs surface markers.^31^, ^32^

Chondrocytes in growth plates are organized into five layers: resting, proliferating, pre‐hypertrophic, hypertrophic and terminal hypertrophic. Resting layer chondrocytes express parathyroid hormone‐related protein (PTHrP). By using Pthrp‐CreER, it is well showed that resting layer chondrocytes are the primary source of growth plate chondrocytes.^33^ And due to the determination of surface markers of SSCs and resting layer chondrocytes, some resting layer chondrocytes in growth plates are considered as a type of SSCs.^15^ Meanwhile, chondrocytes in the resting and proliferative layers actively divide and express high levels of COL2A1.^32^ Pre‐hypertrophic chondrocytes express Ihh, Parathyroid hormone 1 receptor (Pth1r) and Myocyte enhancer factor 2c (Mef2c), while hypertrophic chondrocytes express Runx2, Ihh, Mef2c and collagen type X alpha 1 chain (COL10A1).^32^, ^34^ As they differentiate into pre‐hypertrophic and hypertrophic chondrocytes, they lose their proliferative capacity and undergo hypertrophy in the cartilage anlagen. Eventually, the increasing volume of chondrocytes contributes to the elongation of bones.^35^

### 2.2 Haematopoietic stem cells differentiate into monocyte/macrophage lineage cells to form osteoclasts, macrophages and synovial macrophages

Haematopoietic stem cells (HSCs) are self‐renewing and multipotent cells that differentiate into various blood cell lineages. They first give rise to multipotent progenitors (MPPs), which undergo lineage commitment. The committed MPPs progressively lose their multipotency and produce oligopotent progenitors, such as common myeloid progenitors (CMPs), megakaryocyte erythrocyte progenitors (MEPs) and common lymphoid progenitors (CLPs).^36^ CMPs further differentiate into monocyte/macrophage lineage cells, eventually generating osteoclasts, macrophage and synovial macrophage. Specific markers can identify each of these cell types. For example, HSCs are Lin^−^ CD34^+^ CD38^−^ CD90^+^ CD45RA^−^, MPPs are Lin^−^ CD34^+^ CD38^−^ CD90^−^ CD45RA^−^ and CMPs are Lin^−^ CD34^+^ CD38 ^+^Interleukin 3 (IL3)RA^low^CD45RA^−^.^37^

#### 2.2.1 Receptor activator of nuclear factor kappa‐Β ligand commits the osteoclast lineage of monocyte/macrophage lineage cells

Osteoclasts are derived from bone marrow‐derived monocyte/macrophage lineage cells that circulate in the bloodstream and migrate to the bone surface in response to chemokines and cytokines.^38^ There, they adhere to the bone and initiate osteoclast differentiation. The formation and completion of the osteoclast lineage depend on the action of the Macrophage colony‐stimulating factor (M‐CSF) and receptor activator of nuclear factor kappa‐Β ligand (RANKL).^39^ RANKL recognizes Receptor activator of nuclear factor kappa‐B (RANK) from osteoclast precursors to promote the maturation and differentiation of osteoclasts. Thus, the absence of either *RANK* or *RANKL* results in severe osteopetrosis.^40^, ^41^ The interaction between RANKL and RANK is inhibited by osteoblast‐produced OPG.^42^, ^43^ Therefore, overexpression of *OPG* causes osteopetrosis, while deletion of *OPG* causes severe osteoporosis. Osteomorphs are newly found as the daughter cells of osteoclasts.^44^ The fission of osteoclasts into osteomorphs regulates bone remodelling when receiving RANKL signalling. Osteomorphs express specific genes to distinguish them from osteoclasts and could fuse later to recycle osteoclasts.

Single‐cell spatiotemporal transcriptomics reveals that osteoclast precursors express specific markers such as Colony‐stimulating factor 1 receptor (Csf1r), Adhesion G protein‐coupled receptor e1 (Adgre1), complement C1q A chain (C1qa), complement C1q B chain (C1qb), complement C1q C chain (C1qc), Allograft inflammatory factor 1 (Aif1), triggering receptor expressed on myeloid cells‐2 (Trem2), complement C3a receptor 1 (C3ar1) and CD11c. In contrast, mature osteoclasts express Acid phosphatase 5 (Acp5), TNF receptor superfamily member 11a (Tnfrsf11a), Nuclear factor of activated T cells 1 (Nfatc1), matrix metallopeptidase 9 (Mmp9) and Ctsk.^45^, ^46^

#### 2.2.2 Macrophages, especially synovial macrophages, are important part of the skeletal microenvironment

Differentiation from monocyte/macrophage lineage cells, macrophages are widely present in different tissues. In the osteoimmunity microenvironment, macrophages participate in inflammatory reactions and bone metabolism processes.^47^ Macrophages' polarity transformation is an important regulatory process. Macrophages have two polarized phenotypes, M1 macrophages promote inflammation, while M2 macrophages differentiate to avoid excessive M1 macrophage effects.^47^ Macrophages not only have immune effects in the bone immune microenvironment but also promote osteogenesis.^48^ At the same time, cytokines secreted by macrophages could promote osteoclast generation.^49^

Synovial macrophages (SM) are synovial lining‐derived macrophages. With surface markers including CD11b, CD68, CD14, CD163 and class II major histocompatibility antigens, SM is similar to other macrophages.^50^ Previous studies have suggested that synovial lining cells are loose, but a study has changed this view. It is the C‐X3‐C Motif Chemokine Receptor 1 (CX3CR1)^+^ lining macrophages that form a dense barrier between the synovial sublining and the joint cavity.^51^ The barrier composed of macrophages blocks inflammatory reactions from joints and expands new horizons for the synovial microenvironment.^51^ Research on the pathological subtypes of SM within the synovial microenvironment is still in its infancy, compared with the well‐described fibroblast‐like synoviocytes (FLS). However, SM is reported to affect the chondrogenesis of synovium MSCs through different polarization phenotypes.^52^ SM also contributes to osteoarthritis development.^53^ Incorporating SM into the pathological process and treatment of osteoarthritis will provide new therapeutic targets for osteoarthritis.

### 2.3 The resident FLS are related to the pathological condition of the joint

FLS are synovial lining‐derived fibroblasts. Under physiological conditions, two to three layers of FLS and SM mainly consist the synovial lining.^50^ FLS generates the synovial fluid containing lubricant and hyaluronic acid to lubricate the joint.^54^ FLS are marketed with PDPN, CD90 and Vimentin.^55^ However, the surface markers of FLS are difficult to determine, and different markers may indicate that FLS is in different positions, performing different functions, and even in different pathological stages.^55^ During rheumatoid arthritis, FLS proliferates excessively and secretes abnormally, accompanied by aggressive phenotypes, which all promote rheumatoid arthritis.^54^ Therefore, controlling the response of FLS to rheumatoid arthritis‐inducing factors may be a possible way to prevent rheumatoid arthritis. Although FLS are frequently reported in the context of rheumatoid arthritis, they also play a role in the pathogenesis of osteoarthritis. Traditionally considered a degenerative disease resulting from wear and tear, osteoarthritis is also exacerbated by inflammation driven by FLS.^56^ The osteoarthritic phenotype of FLS is regulated by multiple pathways, including glucose metabolism.^57^, ^58^, ^59^, ^60^ Consequently, targeting these pathways may mitigate the FLS's contribution to the progression of osteoarthritis.

### 2.4 Neural crest stem cells differentiate into osteoblasts and chondrocytes to form most of the craniofacial skeleton

Neural crest stem cells (NCSCs) are a multipotent cell type originating from the neural crest and migrating to various locations in the body. They produce various cell types, including melanocytes, osteoblasts, chondrocytes and smooth muscle cells.^61^ Cranial NCSCs are particularly important for forming the craniofacial skeleton, as they are directed by twist family bHLH transcription factor 1 (*Twist1*) and differentiate into mesenchymal lineages that generate most of the cranial bones.^62^ *FGF8* and homeotic genes are key regulators of NCSC lineage commitment and proliferation during craniofacial development.^63^ Single‐cell RNA sequencing of NCSCs reveals that they undergo a series of binary decisions that lead to mesenchymal specification, which is marked by the expression of paired related homeobox 1 (Prrx1), paired related homeobox 2 (Prrx2) and Twist1.^62^

BMP, FGF, Wnt, Hippo, Notch, Hedgehog, TGF‐β and other pathways regulate the osteoblast differentiation and calvarial bone formation of NCSCs.^64^ Non‐canonical Jagged canonical Notch ligand 1 (JAG1)‐NOTCH1 signalling directs the osteoblast commitment of mesenchymal NCSCs and promotes the repair of craniofacial defects.^65^ NCSC‐derived osteoblasts produce most of the craniofacial skeleton through intramembranous ossification.

**FIGURE 1:**
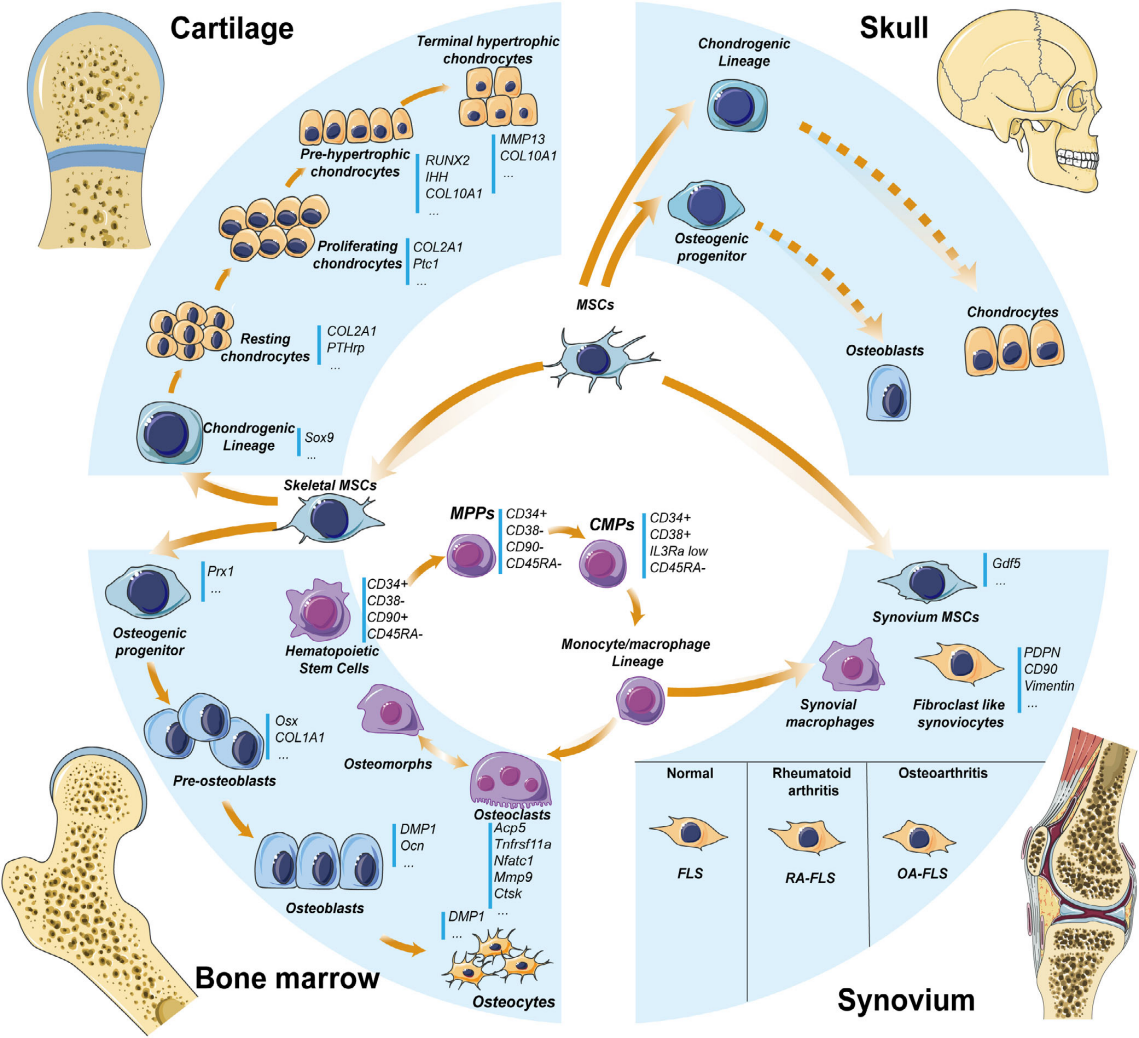
The bone cell populations constitute the main picture of bone homeostasis. They are mostly in four tissues, namely bone marrow, cartilage, synovium and skull. Different stem cells and their differentiated cells in these four tissues are indicated by arrows showing the direction of differentiation. Some cell surface markers are also listed next to the corresponding cells.

*Sox9* also regulates the chondrocyte‐lineage commitment and differentiation of NCSCs by regulating chondrocyte‐specific genes.^66^, ^67^ *Sox9* deletion in NCSCs abolishes their chondrocyte‐lineage potential and causes the absence of NCSC‐derived cartilages and endochondral bones (Figure 1).^68^

## 3 THE HIPPO SIGNALLING PATHWAY REGULATES BONE DEVELOPMENT AND HOMEOSTASIS

The Hippo signalling pathway is a serine/threonine kinase cascade regulating numerous biological processes.^69^, ^70^ It is activated by cellular cues such as communication and signalling between cells, mechanical forces and oxidative stress.^4^, ^69^ The activation involves mammalian sterile 20‐like protein kinases (MST1/2) forming a complex with the scaffold protein Salvador homologue 1 (SAV1) and phosphorylating the MOB domain kinase activator 1 (MOB1A/B), which then activates the large tumour suppressor kinases (LATS1/2).^69^ LATS1/2 inhibit the downstream transcriptional co‐activators YAP and TAZ by phosphorylating them and either confining them to the cytoplasm or targeting them for degradation.^71^, ^72^ If the Hippo signalling pathway gets deactivated, the nuclear translocation of unphosphorylated YAP and TAZ allows them to interact with transcription factors including TEA domain transcription factors 1–4 (TEAD1‐4) to initiate gene transcription.^73^ The Hippo signalling pathway thus modulates fundamental cellular functions concerning organ size and tumorigenesis, such as proliferation, apoptosis, differentiation and stemness.

### 3.1 The Hippo signalling pathway regulates the lineage commitment of MSCs

The Hippo signalling pathway, particularly *YAP/TAZ*, regulates the lineage commitment of MSCs.^74^, ^75^ *YAP/TAZ* could increase the lineage commitment of osteoblasts, rather than adipogenesis.^75^, ^76^ Shear stress and extracellular matrix (ECM) affect the translocation of YAP/TAZ in MSCs, leading to nuclear localization of YAP/TAZ, which activates osteoblast‐lineage genes in MSCs.^77^, ^78^ Targeting these mechanical forces can shift the commitment towards adipogenesis and chondrogenesis.^77^ The osteogenesis of PSCs is enhanced by the upregulation of *BMP*, which is induced by the nuclear translocation of YAP during the fracture healing process.^19^

#### 3.1.1 The Hippo signalling pathway regulates the differentiation and proliferation of osteoblasts to produce different effects

*YAP/TAZ* not only maintains the stemness of MSCs but also commits its osteoblast lineage.^79^ Thus, *TAZ* deletion and *YAP* haploinsufficiency in mesenchymal lineages and osteoblast precursors enhance osteoblast‐lineage commitment and bone mass.^80^ This involves downregulating YAP and TAZ and upregulating *Runx2*, *Osx* and collagen type I alpha I chain (*COL1A1*).^80^, ^81^ Controlled by *Sox2*, *YAP* independently influences the lineage commitment of MSCs, favouring adipogenesis over osteogenesis.^79^ Interestingly, Vestigial‐like family member 4 (VGLL4) can directly counteract the inhibitory effect of TEAD on *Runx2* and promote osteoblast‐lineage differentiation. And this inhibition is independent of YAP, which reveals that VGLL4 and TEADs' osteogenic functions are independent of the Hippo signalling pathway.^82^ Conversely, *YAP/TAZ* promotes the proliferation of osteoblasts. *YAP/TAZ* deletion in osteoblasts and osteocytes causes bone defects and bone mass loss.^75^, ^81^, ^83^

The Mst1/2‐Glut1‐Adenosine 5′‐monophosphate‐activated protein kinase (AMPK) axis also promotes osteoblast‐lineage differentiation independently of the Hippo cascade and YAP/TAZ activities.^84^ Moreover, the calcitonin gene‐related peptide (CGRP)‐*YAP*‐M2 macrophage axis regulates osteoblasts through osteoimmunity. M2 macrophages increase the expression of *ALPL*, *Runx2* and *Osx*, indicating enhanced osteogenic ability.^85^

#### 3.1.2 The Hippo signalling pathway, with a particular focus on YAP/TAZ, regulates the proliferation and differentiation of chondrocytes

*YAP* and *TAZ* are essential regulators of chondrocyte proliferation and differentiation during skeletal development and bone remodelling.^86^, ^87^ *YAP* promotes the proliferation of chondrocytes but later inhibits the maturation of chondrocytes by regulating *Sox6* and *COL10A1*.^87^ Another study also found that *YAP* controlled the proliferation of synovium MSCs and contributed to the differentiation of synovium MSCs into chondrocytes.^7^ *TAZ* promotes both the proliferation and the maturation of chondrocytes and associates with *Sox5* to regulate chondrocyte marker genes.^86^ By knocking out *MOB1A/B* in chondrocytes, *YAP*/*TAZ* activation reduces *Sox9*, *Sox5* and *Sox6* expressions, inhibits the proliferation and maturation of chondrocytes, and causes chondrodysplasia.^88^ ChIP and other experiments resulted in the binding of the YAP/TEAD complex with the *Sox9* promoter and showed converse roles of YAP/TAZ in regulating the proliferation and maturation of cells.^88^ However, impressive in vivo research illustrated that the double knockout of *YAP* and *TAZ* in chondrocytes leads to lethal skeletal deformities.^89^ By deleting the *YAP/TAZ* completely, the expression of *Ctgf* and *Cyr61* could be reduced, but *Sox9* or *COL10A1* would not be affected.^89^ This study considers *YAP/TAZ* as the regulator of morphology but not proliferation, which explains the different phenotypes in vitro and in vivo.^89^ SET domain containing 7 (*SETD7*) modulates the differentiation and glycolysis of chondrocytes under hypoxia through the Hippo signalling pathway and HIF‐1α.^90^

How *YAP/TAZ* regulates *Sox5*, *Sox6*, *Sox9* and other chondrocyte genes in different stages lacks universal staging and evaluation standards, which leads to confusion and mistakes. Similarly, in the chondrogenesis of synovium MSCs, *YAP* can either stimulate or inhibit chondrocyte differentiation and proliferation.^12^ To understand how the Hippo signalling pathway controls chondrocyte proliferation and lineage commitment during bone formation and healing, further research is needed. Although the specific regulatory mechanism remains to be discussed, the Hippo signalling pathway, with a particular focus on *YAP*/*TAZ*, regulates the lineage commitment and proliferation of chondrocytes.

### 3.2 The Hippo signalling pathway regulates how HSCs proliferate, mature and differentiate

The Hippo signalling pathway controls the proliferation and maturation of HSCs. Mechanical forces‐activated *YAP* or activation of *YAP* by knocking out *LATS1/2* both promote the proliferation, maturation and differentiation of HSCs.^91^ *YAP* overexpression in haematopoietic lineages maintains normal HSCs physiology, while *YAP*/*TAZ* deletion disrupts HSCs regulation.^92^ The Scribble/YAP/TAZ complex modulates the activity of cell division cycle 42 (*Cdc42*) and the lineage commitment of HSCs, affecting HSCs function.^92^ *Mst1/2* deletion also impairs HSCs, increasing the production of HSCs‐derived mature cells such as CMPs, which later produce osteoclasts.^93^

#### 3.2.1 The Hippo signalling pathway regulates osteoclastogenesis through various mechanisms

*MST2* deletion promotes osteoclastogenesis through the RANKL pathway, leading to bone mass loss.^94^ The knockout and conditional knockout of *TAZ* in osteoclasts reveal that TAZ interacts with transforming growth factor β‐activated kinase‐1 (TAK1) to inhibit NF‐κB signalling and osteoclastogenesis.^95^ The 11β‐HSD1/Hippo axis also enhances osteoclastogenesis and causes osteoporosis.^96^ YAP/TEAD/activator protein 1 (AP‐1) complex activates *Nfatc1*, *Ctsk* and tartrate‐resistant acid phosphatase (*TRAP*), while *YAP* inhibition reduces YAP/TEAD/AP‐1 complex and NF‐κB signalling, resulting in fewer osteoclasts.^97^ Moreover, *TEAD1* downregulation decreases *OPG* activity and stimulates osteoclast‐lineage differentiation.^98^ Although osteomorphs have just been discovered, their recycling status as osteoclasts has updated views on bone remodelling.^44^ There are numerous cell fate regulation, fission and fusion processes involved in the recycling of osteoclasts as osteomorphs, so Hippo, especially *YAP*/*TAZ*, may once again play its regulatory role in these processes.

#### 3.2.2 The Hippo signalling pathway maintains bone homeostasis by regulating the polarization of macrophages, especially SM

Macrophages are versatile immune cells that can undergo polarization into M1 and M2 phenotypes in response to various stimuli. *YAP* plays a key role in regulating macrophage polarization, but its effects are context‐dependent and sometimes contradictory. *YAP*/*TAZ* activation in renal macrophages enhances their M2 phenotype and contributes to renal fibrosis.^99^ In a liver injury model treated with MSCs, the interaction between YAP and β‐catenin in the macrophage nucleus leads to M2 polarization.^100^ However, *YAP* can also promote M1 polarization of macrophages, which is typically associated with tissue damage and pro‐inflammation. For instance, *YAP* stimulates M1 polarization and IL‐6 production in macrophages during inflammatory bowel disease pathogenesis.^101^ The underlying mechanisms of how *YAP* switches between M1 and M2 polarization are not fully understood but may involve different upstream signals, downstream targets and epigenetic modifications. In the existing studies of bone, *YAP* is temporarily considered to promote M2 polarization of macrophages in bone immune microenvironment.^85^, ^102^ In addition to the molecular factors, mechanical forces can also modulate *YAP* activity and macrophage polarization. It has been shown that macrophages on soft materials have reduced nuclear localization of YAP and exhibit an M2 phenotype than those on stiff materials.^102^ This suggests that *YAP* senses the ECM and regulates the inflammatory response of macrophages accordingly.

The role of *YAP* in SM polarization is still unknown, but it may have important implications for the development and treatment of joint diseases.^103^ SM are located in the synovial membrane that surrounds the joint cavity, and their polarization state can affect joint homeostasis and inflammation. Elucidating how *YAP* regulates SM polarization may provide new insights into the pathophysiology and therapy of joint disorders.

### 3.3 *YAP* / *TAZ* regulates the cellular properties and activities of FLS in both osteoarthritis and rheumatoid arthritis

*YAP*/*TAZ* modulates the phenotype and function of FLS in osteoarthritis and rheumatoid arthritis. FLS are key mediators of joint inflammation and destruction in these diseases. In rheumatoid arthritis, *YAP* enhances the expression of pro‐inflammatory and matrix‐degrading genes in FLS, whereas *YAP* knockout attenuates inflammatory arthritis in mice.^104^ IL‐6, a cytokine implicated in rheumatoid arthritis pathogenesis, stimulates YAP activity and promotes its interaction with Snail, a transcription factor that drives FLS invasiveness, via Jak signalling.^105^ *YAP*/*TAZ* regulates FLS migration, the feature of FLS aggressiveness. Knocking out *YAP*/*TAZ* enhances autophagy and reduces rheumatoid arthritis FLS migration.^106^ Interestingly, Vestigial‐Like Family Member 3 (*VGLL3*), another transcriptional co‐activator of the Hippo pathway, suppresses *TAZ* expression and type I interferon (IFN) production in rheumatoid arthritis FLS.^107^ This suggests that *VGLL3* may have a distinct role in modulating the Hippo pathway in FLS. The lineage origin of FLS is gradually being explored, and the effects of *YAP*/*TAZ* on FLS differentiation and commitment are largely unknown.^108^ The function of *YAP*/TAZ in determining the cell fate of FLS may be clarified by future research, which could also identify new ways to treat osteoarthritis and rheumatoid arthritis.

### 3.4 The migration, setting and lineage commitment of NCSCs are regulated by the Hippo signalling pathway

The Hippo signalling pathway modulates the migration, setting and lineage specification of NCSCs.^109^, ^110^ *YAP*/*TAZ* deletion in cranial NCSCs causes craniofacial defects. These craniofacial defects are associated with disrupted mesenchymal lineages and mandibular haemorrhage in embryos. The YAP‐TEAD complex may involve these phenotypes through forkhead box C1 (*Foxc1*).^110^

The activation of *YAP*/*TAZ* enhances the osteoblast‐lineage commitment. The FoxO6‐LATS1‐YAP axis regulates osteoblast differentiation.^111^ Double knockout of *YAP*/*TAZ* in NCSCs increases the expression of chondrocyte‐specific genes and transforms the NCSCs commitment from osteoblasts to chondrocytes. *YAP*/*TAZ* participates in this process by interacting with β‐catenin.^112^ In osteosarcoma's malignant and metastatic progression, Foxc1 activates the HOXA transcript at the distal tip (*HOTTIP*) to induce LATS2 expression, thereby increasing YAP phosphorylation.^113^

**FIGURE 2:**
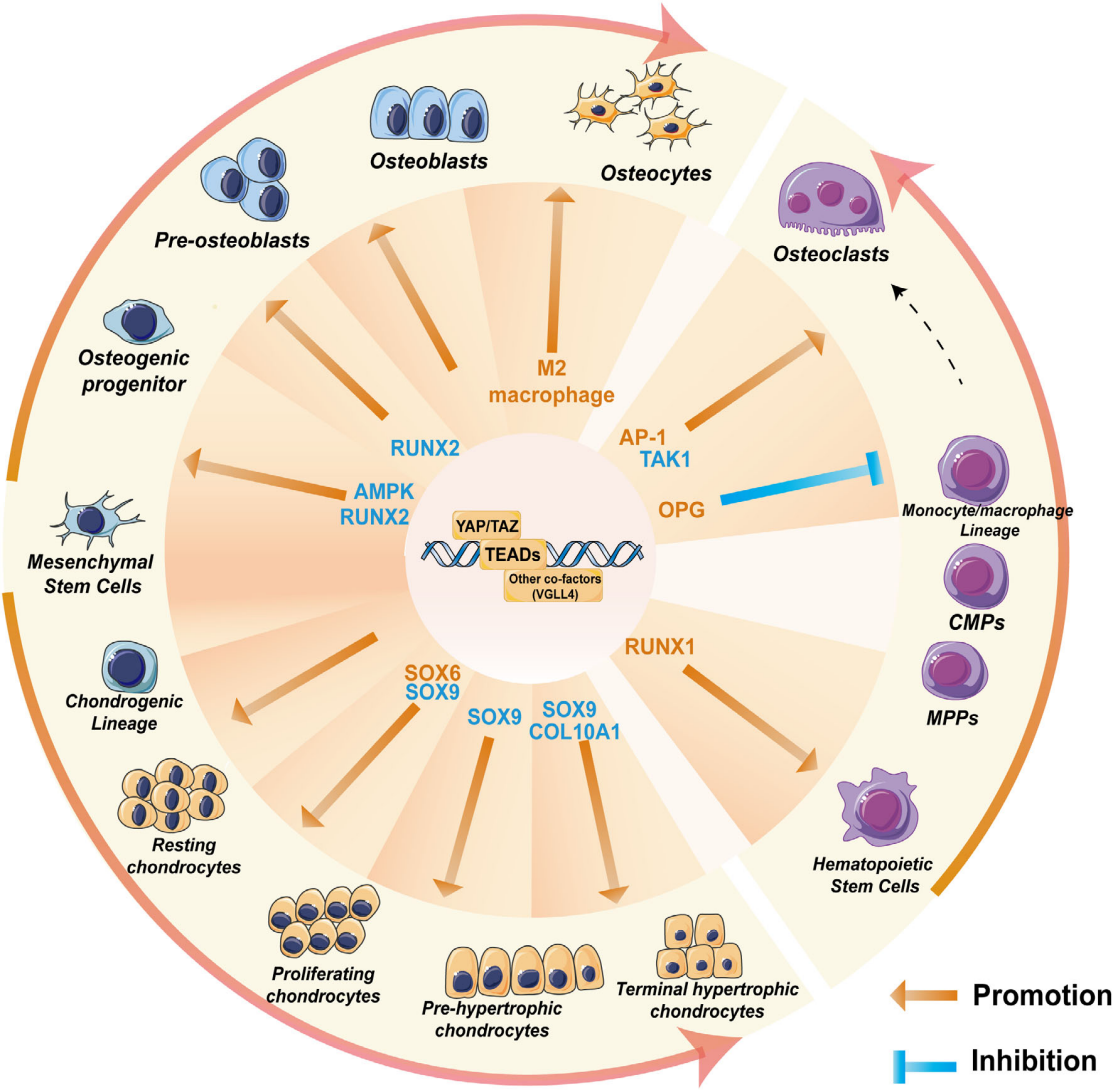
YAP/TAZ exert extensive regulation on the proliferation and differentiation of various cell types in bone homeostasis. However, YAP/TAZ have different, and sometimes opposite, effects on the same lineage cells at different stages of differentiation. This is achieved by modulating a large number of transcription factors. The outer arrows in the figure indicate the direction of differentiation. The inner arrows indicate promotion or inhibition.

The double knockdown of *YAP*/*TAZ* in NCSCs enhances chondrocyte‐lineage commitment (Table 1). These cells express more Sox9 and COL2A1 and less Runx2 and Osx.^112^ FoxO6 directly induces LATS1 to phosphorylate YAP in NCSCs. Consequently, *FoxO6* ^*−/−*^ neonates show delayed endochondral ossification (Figure 2).^111^

**TABLE 1:** Mice models about the Hippo signalling pathway and bone cells.

Mice model‐related genes	The target cells or tissues	Genotype	Description of genotype	Target	Phenotype	Reference
Rb1	MSCs	Prrx1‐Cre; Rb1 floxp/floxp	Rb1 CKO in BMSCs	YAP; Glut1, OPG	Increased bone mass and impaired osteoclastogenesis	114
NF2	Haematopoietic compartment	Mx1‐Cre; NF2 floxp/floxp	NF2 CKO in mouse haematopoietic compartment	‐	Trabecular bone and osteoblast number increase	115
Mst1/2	MSCs	Dermo1‐Cre; Mst1 floxp/floxp; Mst2 floxp/floxp	Mst1/2 dCKOs in MSCs	Col2α1; Col10α1	Cartilaginous callus formation was significantly impaired	87
	Osteoblast precursor cells	Osx‐Cre; Mst1 floxp/floxp; Mst2 floxp/floxp	Mst1/2 dCKOs in osteoblast precursor cells	AMPK‐SMURF1‐RUNX2 axis	A mild but significant reduction of skeletal size including clavicles, forelimbs and hindlimbs	84
	Mature osteoblasts	hOC‐Cre; Mst1 floxp/floxp; Mst2 floxp/floxp	Mst1/2 dCKOs in mature osteoblasts	AMPK‐SMURF1‐RUNX2 axis	Trabecular bone mass was significantly reduced	84
	Chondrocytes	Col2α1‐Cre; Mst1 floxp/floxp; Mst2 floxp/floxp	Mst1/2 dCKOs in chondrocytes	YAP; MMP13	The integrity of articular cartilage of the Mst1f/f; Mst2f/f; Col2a1‐Cre mutant mice were maintained significantly better than that of the control group under both Anterior Cruciate Ligament Transection and Destabilization of the Medial Meniscus surgical conditions	84
	‐	Mst2 −/−	Mst2 KO	NF‐κB pathway	Bone loss; osteoclasts were significantly increased both in size and numbers, whereas osteoblast numbers were considerably reduced	94
Lats1/2 and Mob1a/b	Neural crest	Wnt1‐Cre2; Lats1 floxp/floxp; Lats2 floxp/floxp	Lats1/2 dCKOs in neural crest	TGFB1‐dependent signalling	Embryonic lethal; craniofacial defects of embryos	116
	Chondrocytes	Col2α1‐Cre; Lats1 floxp/floxp; Lats2 floxp/floxp	Lats1/2 dCKOs in chondrocytes	‐	Developmental abnormalities caused by defects in cartilage	89
	Chondrocytes	Col2a1‐CreERT; Mob1a flox/flox; Mob1b−/−	Mob1a CKO in chondrocytes and Mob1b KO	YAP/TAZ; Sox9	Chondrodysplasia; proliferation, differentiation/maturation inhibition of chondrocytes	88
	Chondrocytes	Col2a1‐CreERT; Mob1a flox/flox; Mob1b−/−; YAP floxp/floxp	Mob1a, YAP CKO in chondrocytes, and Mob1b KO	Sox9	The defects in the lengths of the growth plates and the long bones and overall body size were all significantly rescued by the knockout of YAP	88
	Chondrocytes	Col2a1‐CreERT; Mob1a floxp/floxp; Mob1b−/−; TAZ floxp/floxp	Mob1a, TAZ CKO in chondrocytes, and Mob1b KO	Sox9	The defects in the lengths of the growth plates and the long bones and overall body size were all significantly rescued by the knockout of TAZ	88
YAP/TAZ	MSCs	Prrx1‐Cre; YAP floxp/+; TAZ floxp/floxp	YAP HET and TAZ CKO in BMSCs	Runx2; Wnt signalling	Increased cancellous bone volume with increased trabecular number and decreased trabecular separation	81
	Palatal shelves MSCs	Osr2‐Cre; YAP floxp/floxp; TAZ floxp/floxp	YAP/TAZ dCKOs in palatal shelves MSCs	NullL of research	Embryonic lethal; embryos had a similar elevation delay	117
	Posterior palatal shelves MSCs	Col2‐Cre; YAP floxp/floxp; TAZ floxp/floxp	YAP/TAZ dCKOs in posterior palatal shelves MSCs	Ibsp; Phex; Loxl4	The elevation defect of the palatal shelves	117
	Gdf5‐lineage cells	Gdf5‐Cre; YAP floxp/floxp	YAP CKO in Gdf5‐lineage cells	‐	Inhibition of synovial hyperplasia caused by Gdf5‐lineage cells after injury	7
	Neural crest	Wnt1‐Cre2; YAP floxp/+; TAZ floxp/floxp	YAP HET and TAZ CKO in neural crest	β‐catenin; Wnt signalling	Partial embryonic lethal; calvaria bone defects including nasal, frontal and interparietal bones	110
	Neural crest	Wnt1‐Cre2; YAP floxp/floxp; TAZ floxp/floxp	YAP/TAZ dCKOs in neural crest	β‐catenin; Wnt signalling	Embryonic lethal; disrupted craniofacial structures of embryos	110
	Neural crest	Wnt1‐Cre2; YAP floxp/floxp; TAZ floxp/+	TAZ HET and YAP CKO in neural crest	β‐catenin; Wnt signalling	Embryonic lethal; disrupted craniofacial structures of embryos	110
	Neural crest	Wnt1‐Cre; YAP floxp/+; TAZ floxp/floxp	YAP HET and TAZ CKO in neural crest	Mitf	Partial embryonic lethal; craniofacial defects	118
	Neural crest	Wnt1‐Cre; YAP floxp/floxp; TAZ floxp/floxp	YAP/TAZ dCKOs in neural crest	Mitf	Embryonic lethal; disrupted craniofacial structures of embryos	118
	Neural crest	Wnt1‐Cre; YAP floxp/floxp; TAZ floxp/+	TAZ HET and YAP CKO in neural crest	Mitf	Embryonic lethal; disrupted craniofacial structures of embryos	118
	Osteoblast precursor cells	Osx1‐Cre; YAP floxp/floxp; TAZ floxp/floxp	YAP/TAZ dCKOs in osteoblast precursor cells	Runx2; Wnt signalling	Perinatal lethality; osteoblast number and mineralizing surface were significantly increased in vertebral cancellous bone of 12‐week‐old female	81
	Osteoblast precursor cells	Osx1‐Cre; YAP floxp/floxp; TAZ floxp/floxp	YAP/TAZ dCKOs in osteoblast precursor cells	Osx, Alp, Bsp; Col10α1, VEGFA; Col1α1, Col1α2	Impaired fracture healing; reduced callus size; allele dose‐dependent manner of defective periosteal development; reduced bone formation	80
	Osteoblast precursor cells	Osx1‐Cre; YAP floxp/+; TAZ floxp/+	YAP/TAZ dHETs in osteoblast precursor cells	Osx, Alp, Bsp; Col10α1, VEGFA; Col1α1, Col1α2	Impaired fracture healing; reduced callus size; allele dose‐dependent manner of defective periosteal development; reduced bone formation	80
	Osteoblast precursor cells	Osx1‐Cre; YAP floxp/+; TAZ floxp/floxp	YAP HET and TAZ CKO in osteoblast precursor cells	Osx, Alp, Bsp; Col10α1, VEGFA; Col1α1, Col1α2	Impaired fracture healing; reduced callus size; allele dose‐dependent manner of defective periosteal development; reduced bone formation	80
	Osteoblast precursor cells	Osx1‐Cre; YAP floxp/floxp; TAZ floxp/+	TAZ HET and YAP CKO in osteoblast precursor cells	Osx, Alp, Bsp; Col10α1, VEGFA; Col1α1, Col1α2	Impaired fracture healing; reduced callus size; allele dose‐dependent manner of defective periosteal development; reduced bone formation	
	Osteoblasts	Dmp1‐Cre; YAP floxp/floxp; TAZ floxp/floxp	YAP/TAZ dCKOs in mature osteoblasts	Axin2	Decreased bone mineral density; decreased bone volume, decreased trabecular number and increased trabecular separation	81
	Chondrocytes	Col2‐CreERT2; YAP floxp/floxp	Yap CKO in chondrocytes	CTGF; MMP13	Reduced cartilage degeneration to ameliorate OA progression	119
	Chondrocytes	Col2α1‐YAP‐Tg/Tg	YAP conditionalOE in chondrocytes	Runx2; Col10α1	Delayed chondrocyte hypertrophy	87
	Chondrocytes	Col2α1‐Cre; YAP floxp/floxp	Yap CKO in chondrocytes	Runx2; Col10α1	The mutant growth plates were progressively longer; mineralization was significantly increased	87
	Chondrocytes	Col2α1‐YAP‐Tg/+	YAP conditionalOE in chondrocytes	MMP13	Under osteoarthritic condition, Col2a1‐Yap1tg/+ transgenic mice displayed remarkably better cartilage integrity	120
	Chondrocytes	Col2α1‐Cre; YAP floxp/floxp; TAZ floxp/floxp	YAP/TAZ dCKOs in chondrocytes of E17.5 pups	CTGF; Cyr61	Defects in cartilage morphology	89
	Chondrocytes	Col2α1‐Cre; nls‐YAP5SA KI/+	YAP KI in chondrocytes of E17.5 pups	CTGF; Col10α1; MMP16	Developmental abnormalities caused by defects in cartilage	89
	Conventional knockout	TAZ−/−	TAZ KO	TAK1/NF‐κB pathway	Osteoporotic phenotype	95
	Osteoclast‐lineage cells	RANK‐Cre. TAZ floxp/floxp	TAZ CKO in osteoclast‐lineage cells	TAK1/NF‐κB pathway	Osteoporotic phenotype	95
Vgll4	Conventional knockout	Vgll4 −/−	Vgll4 KO	TEAD; Runx2; Alp; Osterix; Col1α1	Dwarfism phenotype with short legs and short clavicles	82
	MSCs	Prrx1‐cre; Vgll4 floxp/floxp	Vgll4 CKO in BMSCs	TEAD; Runx2; Alp; Osterix; Col1α1	Dwarfism; the membranous ossification of the skull and clavicle was also impaired	82
	Osteoblast precursor cells	Osx‐cre; Vgll4 floxp/floxp	Vgll4 CKO in osteoblast precursor cells	TEAD; Runx2; Alp; Osterix; Col1α1	Dwarfism; the membranous ossification of the skull and clavicle was also impaired	82

Abbreviations: AMPK, adenosine 5′‐monophosphate‐activated protein kinase; BMSC, bone marrow mesenchymal stem cells; MMP, matrix metalloproteinase; MSCs, mesenchymal stem cells; NF2, neurofibromin 2; OPG, osteoprotegerin; TAK1, transforming growth factor β‐activated kinase‐1; TAZ, transcriptional co‐activator with PDZ‐binding motif; TEAD, TEA domain; YAP, yes‐associated protein.

## 4 THE INTERACTIONS OF THE HIPPO SIGNALLING PATHWAY WITH OTHER PATHWAYS IN BONE DEVELOPMENT AND HOMEOSTASIS

### 4.1 Wnt and Hippo signalling pathways regulate the development and differentiation of mesenchymal and skeletal lineages

The Wnt signalling pathway, comprising β‐catenin dependent and β‐catenin independent signalling, regulates embryonic development, tissue morphology, and stem cell differentiation.^121^

The canonical signalling is activated by the connection of Wnt ligands and frizzled (FZD), resulting in the stabilization and nuclear translocation of β‐catenin, which modulates the expression of Wnt target genes.^122^ The non‐canonical branch is activated by Wnt ligands, directly triggering signalling cascades. This branch includes the WNT‐planar cell polarity (PCP) and the WNT‐Ca^2+^ pathways.^123^

Wnt and Hippo signalling pathways interact and coordinate to regulate biological processes. YAP can regulate and bind to β‐catenin in the nucleus.^19^, ^124^ Wnt3A stabilizes TAZ, which mediates Wnt signalling.^125^ VGLL4 disrupts the transcription factor 4 (TCF4)/TEAD4 complex and inhibits *TCF4*, *Axin2*, cyclin D1 (*CCND1*) and *Myc* without affecting the nuclear localization of YAP and β‐catenin.^126^

The Hippo signalling pathway can modulate the Wnt signalling pathway positively and negatively by various mechanisms, which may depend on the different responses of YAP/TAZ to Wnt signalling. This crosstalk finally promotes the lineage commitment of MSCs. For example, exosomal Wnt5A regulates *YAP* to induce a tumour‐promoting lineage of MSCs.^127^ Wnt3A/YAP/TEAD and Wnt3A/PP1A/TAZ axes promote the osteoblast‐lineage differentiation of MSCs.^128^, ^129^ YAP/β‐catenin complex enhances osteogenesis and inhibits adipogenesis in MSCs and osteoblasts.^75^ *Wnt1* also increases bone formation through *YAP* and *BMP* in osteoblasts.^130^ YAP activates β‐catenin and Wnt signalling to reduce chondrogenesis and hypertrophic differentiation.^131^ However, another study shows that Runt‐related transcription factor 1 (*Runx1*) deletion decreases YAP but activates β‐catenin, leading to articular cartilage degradation.^132^ These opposite effects may be due to the different roles of *YAP* in different stages of chondrocytes and the indirect regulation of β‐catenin by YAP in *Runx1* ^*−/−*^ mice. *YAP* also regulates β‐catenin to facilitate the migration of NCSCs.^133^ In summary, Wnt and Hippo signalling pathways coordinately regulate the proliferation and lineage commitment of mesenchymal and skeletal lineages, but the underlying molecular mechanisms are still elusive. These crosstalks to be discovered may offer new opportunities and approaches for treating skeletal disorders.

### 4.2 The interplay of TGF‐β signalling and Hippo signalling pathway in the development and differentiation of stem cells and osteoblasts

TGF‐β superfamily members, such as TGF‐βs, BMPs, activins, growth differentiation factors (GDFs) and müllerian inhibiting substance (MIS), regulate the differentiation of osteoblasts and chondrocytes.^134^, ^135^ Osteoblast‐specific deletion of TGF‐β type II receptor (*TβRII*) increases bone mass.^136^ Several studies have revealed crosstalk between the Hippo signalling pathway and TGF‐β signalling.^137^ For example, BMP‐2/Smad1/5/8/YAP/TAZ axis commits osteoblast lineage in myoblasts.^138^ TGF‐β signalling activates hexokinase 2 (*HK2*) to enhance glycolysis and lung fibrosis through YAP/TAZ.^139^ However, the molecular mechanism of HK2 and YAP/TAZ interaction remains unclear. TGF‐β signalling commits cell lineages of MSCs.^140^ YAP/TAZ modulates BMP and TGF‐β's transcriptional responses by interacting with SMAD1/5.^141^ YAP mediates the BMP‐induced migration of NCSCs.^133^ Another study shows that LATS1/2‐regulated YAP modulates the epithelial‐to‐mesenchymal transition in NCSCs driven by TGF‐β signalling components.^116^ All these findings demonstrate the complex and dynamic interactions between the Hippo signalling pathway and TGF‐β signalling in regulating the differentiation and function of mesenchymal and skeletal lineages.

### 4.3 The Hippo signalling pathway interacts with NF‐κB pathways to regulate bone formation and homeostasis

The regulation of bone metabolism by YAP is achieved through the reduction of the NF‐κB signalling pathway activation (Table 2).^142^ In addition, YAP can decrease the transmission of the NF‐κB signalling pathway by directly inhibiting the substrate accessibility of TAK1.^120^ The molecular and physiological consequences of the interplay between Hippo and NF‐κB pathways in bone formation and homeostasis require further investigation.

**TABLE 2:** Mice models about the Hippo signalling pathway crosstalk with other pathways to regulate bone homeostasis.

Genotype	Description of genotype	The protein crosstalk with Hippo	The Hippo members crosstalk with other proteins	Target	Phenotype	Reference
Prrx1Cre; Piezo1fl/fl	Piezo1 CKO in MSCs	Piezo1	YAP	COL2A1, COL9A1	Osteoporosis	143
Prrx1‐Cre; Piezo1f/f	Piezo1 CKO in MSCs	Piezo1	YAP	NFATc1/YAP1/β‐catenin complex	Severe skeletal defects	144
Prrx1‐Cre; Piezo1f/f; Piezo2f/f	Piezo1/2 dCKOs in MSCs	Piezo1/2	YAP	NFATc1/YAP1/β‐catenin complex	Severe skeletal defects	124
WT with piezo70 shRNA	Piezo1 KO	Piezo1	YAP	YAP1/β‐catenin complex	Union was delayed, and callus mineralization was impaired	19
LysM‐Cre; YAP floxp/floxp	YAP CKO in macrophages	ROS	YAP	GPX4	Negatively affected bone defect repair; delayed bone defect repair process and looser regenerated new bone after 2 weeks	145
FoxO6 −/−	FoxO6 KO	FoxO6	Lats1	YAP	Delayed ossification and a flat skull, while adult heads are expanded anteriorly	111
11β‐HSD1 KI/KI	11β‐HSD1 OE	11β‐HSD1	YAP	PDGF‐BB	Osteoporosis	96
Ctsk‐Cre; 11β‐HSD1 floxp/floxp	11β‐HSD1 CKO in osteoclasts	11β‐HSD1	YAP	PDGF‐BB	Rescued osteoporosis	96
Col2α1‐Cre; Runx1 floxp/floxp	Runx1 CKO in chondrocytes	‐	‐	‐	Exacerbated articular cartilage damage after DMM surgery	132
Col2α1‐CreER; Runx1 floxp/floxp	Runx1 CKO in chondrocytes after birth	Runx1	Lats2	YAP; Col2α1	OA‐like phenotype	83
Dmp1‐Cre; YAP floxp/floxp; TAZ floxp/floxp	YAP/TAZ dCKOs in mature osteoblasts	TGF‐β signalling	YAP/TAZ‐TEAD	CYR61; CTGF	Reduced femoral length at P84; reduced bone volume fraction, trabecular number and thickness and increased trabecular spacing and structural model index; reduced cortical thickness and bone area in mid‐diaphyseal femoral cortical bone	83
Col2α1‐Cre; YAP floxp/floxp	YAP CKO in chondrocytes	TAK1	YAP	NF‐κB pathway	Cartilage degradation was significantly more severe than that of the control mice	120
Ocn‐Cre; YAP floxp/floxp	YAP CKO in osteoblast‐lineage cells	β‐catenin	YAP	YAP1/β‐catenin complex	Decreased bone formation, and trabecular bone loss	75
Wnt1‐Tg	Wnt1 OE	Wnt1	YAP	BMP2	Increases bone formation in the fracture callus	130
Osx1‐Cre; YAP floxp/floxp; TAZ floxp/floxp	YAP/TAZ dCKOs in osteoblast precursor cells	β‐catenin	YAP	Osx, Alp, Bsp; Col10α1, VEGFA; Col1α1, Col1α2	Impaired fracture healing; reduced callus size; allele dose‐dependent manner of defective periosteal development; reduced bone formation	80
Osx1‐Cre; YAP floxp/+; TAZ floxp/+	YAP/TAZ dHETs in osteoblast precursor cells	β‐catenin	YAP	Osx, Alp, Bsp; Col10α1, VEGFA; Col1α1, Col1α2	Impaired fracture healing; reduced callus size; allele dose‐dependent manner of defective periosteal development; reduced bone formation	80
Osx1‐Cre; YAP floxp/+; TAZ floxp/floxp	YAP HET and TAZ CKO in osteoblast precursor cells	β‐catenin	YAP	Osx, Alp, Bsp; Col10α1, VEGFA; Col1α1, Col1α2	Impaired fracture healing; reduced callus size; allele dose‐dependent manner of defective periosteal development; reduced bone formation	80
Osx1‐Cre; YAP floxp/floxp; TAZ floxp/+	TAZ HET and YAP CKO in osteoblast precursor cells	β‐catenin	YAP	Osx, Alp, Bsp; Col10α1, VEGFA; Col1α1, Col1α2	Impaired fracture healing; reduced callus size; allele dose‐dependent manner of defective periosteal development; reduced bone formation	80

Abbreviations: BMP, bone morphogenetic protein; MSCs, mesenchymal stem cells; NF2, neurofibromin 2; ROS, reactive oxygen species; TAK1, transforming growth factor β‐activated kinase‐1; TAZ, transcriptional co‐activator with PDZ‐binding motif; TEAD, TEA domain; YAP, yes‐associated protein; TGF‐β Transforming growth factor beta; YAP, yes‐associated protein.

## 5 MECHANICAL FORCES AND MECHANOSENSORS AS TWO REVISED PERSPECTIVE MODULATE BONE HOMEOSTASIS BY REGULATING THE HIPPO SIGNALLING PATHWAY

Mechanical forces modulate cellular functions by activating mechanosensors, which transduce the signals to downstream pathways. Among the mechanosensors, YAP and TAZ are key factors that link mechanical cues to biological responses.^146^, ^147^ They are located in the nucleus when cells adhere to rigid ECM but are cytoplasmic and inactive on soft ECM. Their localization and activity are regulated by both Hippo‐dependent and ‐independent mechanisms.^146^ Various types of mechanosensors, such as integrins, G protein‐coupled receptors and Piezo1 ion channels, can influence the Hippo pathway in different ways. We will discuss how these mechanosensors modulate the Hippo pathway in a categorized manner.

### 5.1 Focal adhesions

FAs are molecular complexes that link cells to the ECM and mediate receptor‐ligand interactions.^148^ They transmit signals from the matrix or other cells to the intracellular environment and connect with the cytoskeleton. FAs participate in mechanical signal transduction and regulate bone homeostasis. Cell adhesion molecules involved in FAs include integrin, cadherin, selectin, IgSF and others.^148^

#### 5.1.1 Integrin

Integrin, as an adhesion molecule, mediates the transmission of mechanical signals. Cells detect mechanical forces by enhancing F‐actin polymerization via integrin‐mediated adhesion. Mechanical forces activate integrin to activate Rho‐GTPases and F‐actin, which increase actin cytoskeleton tension and nuclear pore dilation, facilitating YAP/TAZ nuclear entry.^149^ Therefore, inhibition of RHO, myosin, RHO‐associated kinase (ROCK), myosin light chain kinase (MLCK) or F‐actin polymerization reduces cellular tension and nuclear localization of YAP/TAZ.^150^, ^151^ F‐actin also modulates c‐Jun N‐terminal kinase (JNK)/LIM domain containing 1 (LIMD1) and Neurofibromin 2 (NF2) to suppress LATS1/2 and consequently elevate YAP.^151^, ^152^ Notably, NF2 sequesters LATS1/2 at the plasma membrane.^152^ The ROCK‐YAP/TAZ axis exemplifies the essential role of YAP/TAZ as mechanotransducers. MMP14 can modulate the shape of MSCs to activate integrin and trigger the Rho/ROCK pathway.^77^ This leads to the nuclear translocation and activation of YAP/TAZ, which promotes the osteogenesis of MSCs. Similarly, targeting Rho/ROCK and YAP/TAZ can affect the lineage commitment of MSCs.^153^, ^154^ Inhibition of Rho/ROCK reduces YAP/TAZ nuclear localization and osteogenic gene expression, such as *BMP*, *Runx2*, *Osx* and *Sox9*.^153^, ^154^ The Rho/ROCK‐YAP/TAZ axis also mediates the M2 polarization of macrophages, which supports osteogenesis.^102^ By activating Src kinase, integrin increases the nuclear localization and activity of YAP/TAZ.^155^ Cerebral cavernous malformation 3 (CCM3) blocks the mechanotransduction of F‐actin to restrain YAP.^156^ CCM3 controls the differentiation of MSCs through this mechanism. AT‐rich interaction domain 1a (ARID1A) and SWItch/Sucrose nonfermentable (SWI/SNF) sequester YAP/TAZ by binding to them and competing with TEADs.^157^ In the nucleus, F‐actin antagonizes ARID1A/SWI/SNF‐YAP/TAZ to promote the nuclear localization of YAP.^157^

#### 5.1.2 Cadherins

Cadherins are a class of transmembrane proteins that rely on calcium to mediate cell–cell interactions. They include epithelial cadherin (E‐cadherin), neural cadherin (N‐cadherin) and vascular endothelial cadherin (VE‐cadherin).^158^ Cadherins often act in opposition to integrins. This antagonism may result from their competitive binding to F‐actin and their shared signalling proteins.^159^ Cadherins and integrins also have opposite roles in regulating the Hippo signalling pathway. Cadherins sequester YAP in the cytoplasm in the absence of mechanical forces, whereas integrins promote their nuclear translocation in response to mechanical forces.^160^

VE‐cadherin interacts with the 14‐3‐3‐YAP complex and prevents its nuclear entry into endothelial cells.^161^ However, epidermal growth factor receptor pathway substrate 8 (EPS8), an actin signalling adapter protein, can disrupt this interaction and enhance YAP nuclear localization and transcriptional activity.^161^ E‐cadherin activates NF2 to inhibit YAP activity in mesenchymal cancer cells, thereby preventing ferroptosis, a form of regulated cell death.^162^ N‐cadherin suppresses YAP activity on soft surfaces but enhances it on hard surfaces, leading to ferroptosis induction.^163^ Cadherins also modulate MSCs differentiation by influencing YAP localization. RGD/integrin and HAVDI/N‐cadherin connections have opposite effects on filamin phosphorylation, a key event for YAP nuclear entry.^164^ In this process, cadherin also partially inhibits integrin mechanotransduction. Phosphorylated filamin drive YAP into the nucleus and stimulate the osteogenesis of MSCs.^164^ N‐cadherin can reverse this process by exporting YAP from the nucleus to the cytoplasm, resulting in reduced expression of osteogenic markers Runx2, alkaline phosphatase (ALP) and osteocalcin (OCN) and loss of MSCs osteogenic lineage commitment.^165^ The antagonistic roles of cadherins and integrins in bone mechanotransduction cannot be fully explained by their competition for cytoskeletal binding and the inhibition of cadherins on integrins. Their differential effects on YAP localization may also contribute to this antagonistic phenomenon.

### 5.2 G protein‐coupled receptors

G protein‐coupled receptors are upstream of the Hippo signalling pathway.^166^ Although we review GPCRs as mechanosensors, only a subset of GPCRs can sense mechanical forces.^167^, ^168^ These mechanosensitive GPCRs modulate the nuclear translocation of YAP/TAZ by controlling actin dynamics through Rho‐GTPases, similar to integrins.^169^, ^170^ Lysophosphatidic acid (LPA)‐activated GPCRs also enhance YAP/TAZ nuclear localization via Rho‐GTPases.^170^, ^171^ The mechanotransduction of GPCRs signalling is regulated by protein kinase A (PKA).^171^

### 5.3 Piezo1

Piezo1 is a kind of mechanically activated cation channel. Deletion of Piezo1 and Piezo2 in osteoblasts results in reduced bone mass and severe skeletal defects.^124^, ^143^ Piezo1 affects YAP/TAZ through various mechanisms. A recent study demonstrates that Piezo1 acts primarily on osteoblasts, while its ablation in osteoclasts does not alter bone mass. Piezo1 regulates YAP localization, which affects type II and IX collagens.^143^ These collagen isoforms then influence osteoclast differentiation. Another study unveils a distinct mechanism of Piezo1 in osteoblasts. Piezo1 triggers Ca^2+^ influx to coordinate the activation of NFAT, YAP and β‐catenin. The NFAT/YAP/β‐catenin complex drives osteoblast differentiation and bone formation.^124^ Similarly, Pizeo1 stimulates the YAP/β‐catenin complex in PSCs, enhancing osteoblast differentiation and bone formation.^19^ After treatment with Piezo1 activator Yoda1, the activated YAP accelerated the healing of mouse fractures. In valve interstitial cells (VICs), Piezo1 activity induces YAP. YAP regulates glutaminase1‐mediated glutaminolysis and subsequently activates *Runx2* and osteogenesis.^172^ Cyclic mechanical tension stimulates YAP through Piezo1 in human endplate chondrocytes.^144^ Then, the activities of YAP and TEAD1 increase the expression of *COL2A1* and *ACAN*.

In summary, YAP and TAZ are versatile mechanotransducers that mediate diverse biological processes in response to mechanical cues. New insights into how YAP/TAZ work at the molecular level may help us regulate tissue homeostasis and avoid diseases that result from mechanical forces.

## 6 AGING FACTORS LEAD TO DYSREGULATION OF THE HIPPO SIGNALLING PATHWAY, WHICH CAUSES THE AGING PHENOTYPES OF BONES

Aging is a multifactorial process that leads to various cellular, tissue and organismal changes, such as altered bone cell number and stem cell differentiation, osteoarthritis and osteoporosis.^143^ Identifying the causal factors of aging and their temporal sequence is a major challenge in aging research.^173^ A review categorized the factors of aging into genomic instability, cell cycle arrest, mitochondrial dysfunction, protein homeostasis loss, metabolic dysregulation and others.^174^ These factors can modulate the rate of aging by either exacerbating or alleviating age‐related damage. The interactions among these factors are complex and dynamic, and some factors can act as both causes and consequences of aging. To avoid ambiguity, we focus on how these aging factors affect the Hippo signalling pathway, which later influences aging phenotypes and dysregulation of bone homeostasis.

The Hippo signalling pathway regulates cellular aging in a cell type‐ and state‐dependent manner. In most cells, the nuclear localization of YAP/TAZ declines with aging.^175^ YAP/TAZ confer a protective role in senescent cells, preventing premature aging.^89^, ^176^ However, this effect can be abolished by verteporfin, which accelerates cellular aging by inhibiting YAP activity.^5^ We review how mechanical cues, oxidative stress, abnormal gene activation, RNA dysregulation and other aging factors modulate the Hippo pathway to induce aging phenotypes and inbalance of bone homeostasis.

### 6.1 Mechanical cues

More and more studies suggest that mechanical cue is an independent aging factor. Some studies suggest that mechanical cue causes genomic instability and affects aging.^6^, ^177^ However, considering that mechanical force directly regulates the Hippo signalling pathway in the aging process and that genomic instability may be an aging phenotype rather than an aging factor, we propose that mechanical force transmission is an independent aging factor. Reduced mechanical transmission in stromal cells leads to accelerated aging phenotypes and downregulation of *YAP*/*TAZ*.^175^ In general, the abnormality of mechanical transmission causes the dysregulation of the Hippo signalling pathway, thereby accelerating the aging phenotype. Another study showed that cell–cell contact in hepatic stellate cells inhibited *YAP* activity, leading to accelerated aging.^178^ However, how mechanical transmission affects bone‐related cells through YAP/TAZ to promote aging phenotype is still lacking in research.

### 6.2 Oxidative stress

Mitochondrial dysfunction is another cause of premature aging, as it generates reactive oxygen species (ROS) that impair the respiratory chain and induce oxidative stress.^174^ Peroxisome‐proliferator‐activated receptor γ co‐activator 1‐α (PGC‐1α) is a key protein that reduces ROS production and prevents mitochondrial dysfunction‐induced aging.^179^ PGC‐1α deficiency leads to less TAZ and increased adipogenic differentiation of MSCs.^179^ Inhibition of the ROS‐Hippo axis can rescue nucleus pulposus cell aging.

### 6.3 Abnormal gene activation

Abnormal signalling pathways, epigenetic genes and oncogenes, such as Ras and CBX4, also contribute to cellular aging by inhibiting proliferation or metabolism. Ras suppresses YAP/TAZ through multiple pathways, causing cells to lose their anti‐aging functions.^180^ Overexpression of YAP can counteract Ras‐induced metabolic disorders and delay aging. Chromobox protein homologue 4 (CBX4) is a protein linked to MSCs aging.^181^ Lentivirus overexpressing CBX4 can alleviate osteoarthritis in mice and reduce senescent cells. The Hippo signalling pathway has been demonstrated to be a downstream effector of CBX4 and respond to its regulation, but the specific regulatory mechanism remains to be elucidated.^182^ Future studies may provide new insights into the Hippo‐mediated response to aging‐related genes.

### 6.4 RNA dysregulation

Long non‐coding RNA (lncRNA) and microRNA (miRNA) are emerging as novel aging factors in bone, as they regulate MSCs proliferation and lineage commitment via the Hippo signalling pathway. Bmncr is a lncRNA that decreases with age and promotes *TAZ* transcriptional activity.^183^ Bmncr knockout mice show accelerated aging, which can be alleviated by overexpressing *TAZ*. MiR‐155‐5p is a miRNA that induces aging and reduces *YAP* in BMSCs.^184^ Transfection of miR‐155‐5p inhibitor can enhance BMSCs proliferation and delay their aging. *YAP* is a downstream target gene of miR‐155‐5p, so knocking down *YAP* inhibits BMSCs proliferation and accelerates BMSCs aging. In another study, aging results in downregulation of *YAP* through the miR‐155‐5p/brain and muscle arnt‐like 1 (Bmal1)/YAP axis, decreasing MSCs differentiation potential and osteoblast‐lineage differentiation. Thus, the activation of the Hippo signalling pathway during aging is demonstrated through YAP/TEAD binding inhibitors Vertepofin and *Bmal1*‐OE. Specifically, even though the Hippo signalling pathway is activated, the miR‐155‐5p/Bmal1/YAP axis has an independent effect on MSCs, independent of the Hippo cascade. Therefore, by regulating the known YAP/TAZ‐related axes, we could rescue bone loss and other aging phenotypes and even achieve de‐senescence. Injecting *Bmal1*‐OE or miR‐155‐5p inhibitor‐treated MSCs into elderly mice using the miR‐155‐5p/Bmal1/YAP axis can reduce YAP expression and alleviate bone loss in elderly mice.^184^

**FIGURE 3:**
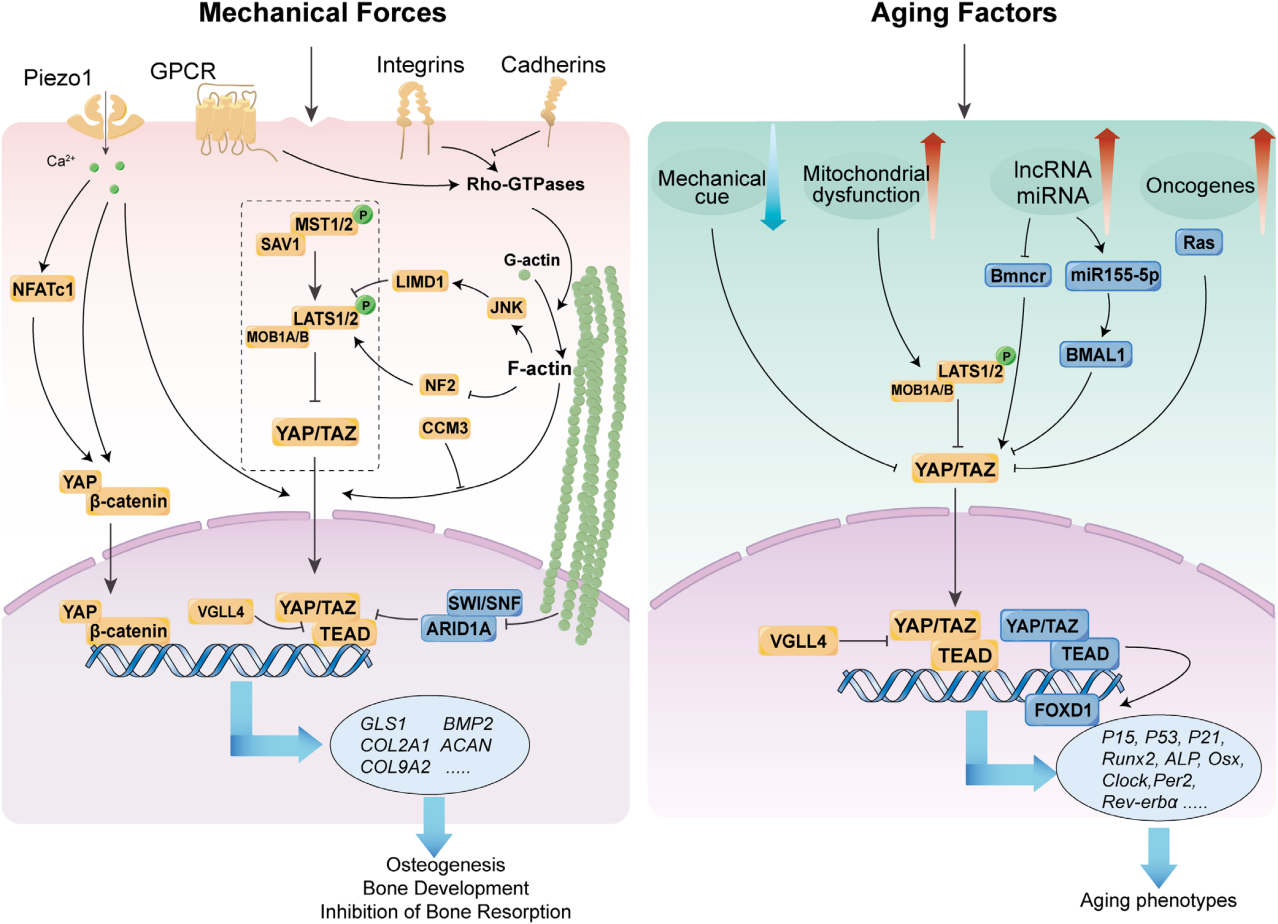
The Hippo signalling pathway regulates bone homeostasis under mechanical forces and aging factors. YAP/TAZ respond to the regulation of various molecules, such as Piezo1, GPCRs, Integrin, Cadherin and thus act as transducers to regulate bone homeostasis. Meanwhile, the Hippo signalling pathway is modulated by multiple aging factors, such as mechanical cues, mitochondrial dysfunction, abnormal RNA and aberrant genes, resulting in the aging phenotypes of the skeleton.

The Hippo signalling pathway is a crucial link between aging factors and aging phenotypes (Figure 3). However, the regulation of this pathway by aging factors remains poorly understood, as most studies have focused on how the pathway affects aging phenotypes. Exploring how aging factors dysregulate the Hippo signalling pathway could uncover novel mechanisms of aging and offer new therapeutic targets.

## 7 DISCUSSION

The Hippo signalling pathway is a complex and critical regulatory network that governs various cellular processes, including cell proliferation, apoptosis, differentiation and stem cell functions.^185^ Although its role in bone homeostasis has been extensively studied, its involvement in chondrogenesis and cartilage homeostasis remains confused. How the Hippo signalling pathway regulates the bone development and homeostasis of skeletal cells reflects its extensive regulation. Meanwhile, the interaction between the Hippo signalling pathway and Wnt, TGF‐β and NF‐κB signalling pathways, commits the skeletal lineages and regulates the proliferation of these cells. More studies are necessary to elucidate the interaction between other pathways and the Hippo signalling pathway.

Lastly, we focus on who regulates the Hippo signalling pathway, especially YAP/TAZ as mechanotransducers, to regulate bone homeostasis. We review that mechanical forces regulate the Hippo signalling pathway through mechanosensors including integrin, cadherins, GPCRs and Piezo1. We also review that aging influences bone development and homeostasis through dysregulating the Hippo signalling pathway. However, what connects aging factors with the Hippo signalling pathway still needs more studies to elucidate.

Further in‐depth understanding of the Hippo signalling pathway and crosstalk between the Hippo signalling pathway and other pathways in bone development and homeostasis is beneficial to treating bone diseases.

## AUTHOR CONTRIBUTIONS

Zuoyun Wang and Jinlong Suo conceived, reviewed and edited the manuscript. Zhengda Li, Junqing Lin and Jing Wu drafted the manuscript. Zhengda Li created the figures and tables. All authors read and approved the final manuscript.

## FUNDING INFORMATION

This research is supported by the National Key R&D Program of China (2020YFA0803201 to Z.W.; 2022YFA1106400 to Z.W.), the National Natural Science Foundation of China (32270886 and 32070827 to Z. W.).

## CONFLICT OF INTEREST STATEMENT

The authors declare that there are no conflict of interests.

## Data Availability

Figures 1 and 2 were modified from Servier Medical Art (http://smart.servier.com/), licensed under a Creative Common Attribution 3.0 Generic Licence (https://creativecommons.org/licenses/by/3.0/).

## References

[1] Jin F , Liu M , Zhang D , Wang X . Translational perspective on bone‐derived cytokines in inter‐organ communications. Innovation. 2023;4:100365. 36624884 10.1016/j.xinn.2022.100365 PMC9823204

[2] Lv X , Gao F , Cao X . Skeletal interoception in bone homeostasis and pain. Cell Metab. 2022;34:1914‐1931. 36257317 10.1016/j.cmet.2022.09.025 PMC9742337

[3] Xie L , Wang G , Wu Y , et al. Programmed surface on poly(aryl‐ether‐ether‐ketone) initiating immune mediation and fulfilling bone regeneration sequentially. Innovation. 2021;2:100148. 34557785 10.1016/j.xinn.2021.100148 PMC8454576

[4] Liu H , Jiang D , Chi F , Zhao B . The Hippo pathway regulates stem cell proliferation, self‐renewal, and differentiation. Protein Cell. 2012;3:291‐304. 22549587 10.1007/s13238-012-2919-3 PMC4728156

[5] Anerillas C , Mazan‐Mamczarz K , Herman AB , et al. The YAP‐TEAD complex promotes senescent cell survival by lowering endoplasmic reticulum stress. Nat Aging. 2023;3:1237‐1250. 37667102 10.1038/s43587-023-00480-4 PMC11369890

[6] Nava MM , Miroshnikova YA , Biggs LC , et al. Heterochromatin‐driven nuclear softening protects the genome against mechanical stress‐induced damage. Cell. 2020;181:800‐817. 32302590 10.1016/j.cell.2020.03.052 PMC7237863

[7] Roelofs AJ , Zupan J , Riemen A , et al. Joint morphogenetic cells in the adult mammalian synovium. Nat Commun. 2017;8:15040. 28508891 10.1038/ncomms15040 PMC5493527

[8] Wang Y , Fang J , Liu B , Shao C , Shi Y . Reciprocal regulation of mesenchymal stem cells and immune responses. Cell Stem Cell. 2022;29:1515‐1530. 36332569 10.1016/j.stem.2022.10.001

[9] Viswanathan S , Shi Y , Galipeau J , et al. Mesenchymal stem versus stromal cells: International Society for Cell & Gene Therapy (ISCT®) Mesenchymal Stromal Cell committee position statement on nomenclature. Cytotherapy. 2019;21:1019‐1024. 31526643 10.1016/j.jcyt.2019.08.002

[10] Zamudio‐Cuevas Y , Plata‐Rodríguez R , Fernández‐Torres J , et al. Synovial membrane mesenchymal stem cells for cartilaginous tissues repair. Mol Biol Rep. 2022;49:2503‐2517. 35013859 10.1007/s11033-021-07051-z

[11] Li P , Fu L , Liao Z , et al. Chitosan hydrogel/3D‐printed poly(ε‐caprolactone) hybrid scaffold containing synovial mesenchymal stem cells for cartilage regeneration based on tetrahedral framework nucleic acid recruitment. Biomaterials. 2021;278:121131. 34543785 10.1016/j.biomaterials.2021.121131

[12] Tao SC , Yuan T , Zhang YL , Yin WJ , Guo SC , Zhang CQ . Exosomes derived from miR‐140‐5p‐overexpressing human synovial mesenchymal stem cells enhance cartilage tissue regeneration and prevent osteoarthritis of the knee in a rat model. Theranostics. 2017;7:180‐195. 28042326 10.7150/thno.17133 PMC5196895

[13] Chan CK , Seo EY , Chen JY , et al. Identification and specification of the mouse skeletal stem cell. Cell. 2015;160:285‐298. 25594184 10.1016/j.cell.2014.12.002 PMC4297645

[14] Chan C , Gulati GS , Sinha R , et al. Identification of the human skeletal stem cell. Cell. 2018;175:43‐56. 30241615 10.1016/j.cell.2018.07.029 PMC6400492

[15] Feng H , Jiang B , Xing W , Sun J , Greenblatt MB , Zou W . Skeletal stem cells: origins, definitions, and functions in bone development and disease. Life Med. 2022;1:276‐293. 36811112 10.1093/lifemedi/lnac048 PMC9938638

[16] Jeffery EC , Mann T , Pool JA , Zhao Z , Morrison SJ . Bone marrow and periosteal skeletal stem/progenitor cells make distinct contributions to bone maintenance and repair. Cell Stem Cell. 2022;29:1547‐1561. 36272401 10.1016/j.stem.2022.10.002

[17] Han Y , Feng H , Sun J , et al. Lkb1 deletion in periosteal mesenchymal progenitors induces osteogenic tumors through mTORC1 activation. J Clin Invest. 2019;129:1895‐1909. 30830877 10.1172/JCI124590 PMC6486357

[18] Debnath S , Yallowitz AR , McCormick J , et al. Discovery of a periosteal stem cell mediating intramembranous bone formation. Nature. 2018;562:133‐139. 30250253 10.1038/s41586-018-0554-8 PMC6193396

[19] Liu Y , Tian H , Hu Y , et al. Mechanosensitive Piezo1 is crucial for periosteal stem cell‐mediated fracture healing. Int J Biol Sci. 2022;18:3961‐3980. 35844802 10.7150/ijbs.71390 PMC9274506

[20] Tsukasaki M , Komatsu N , Negishi‐Koga T , et al. Periosteal stem cells control growth plate stem cells during postnatal skeletal growth. Nat Commun. 2022;13:4166. 35851381 10.1038/s41467-022-31592-x PMC9293991

[21] Komori T . Regulation of proliferation, differentiation and functions of osteoblasts by Runx2. Int J Mol Sci. 2019;20:1694. 30987410 10.3390/ijms20071694 PMC6480215

[22] Komori T . Whole aspect of Runx2 functions in skeletal development. Int J Mol Sci. 2022;23:5776. 35628587 10.3390/ijms23105776 PMC9144571

[23] Chen Q , Shou P , Zheng C , et al. Fate decision of mesenchymal stem cells: adipocytes or osteoblasts? Cell Death Differ. 2016;23:1128‐1139. 26868907 10.1038/cdd.2015.168 PMC4946886

[24] Zhu S , Ehnert S , Rouß M , et al. From the clinical problem to the basic research‐co‐culture models of osteoblasts and osteoclasts. Int J Mol Sci. 2018;19:2284. 30081523 10.3390/ijms19082284 PMC6121694

[25] Shi Y , He G , Lee WC , McKenzie JA , Silva MJ , Long F . Gli1 identifies osteogenic progenitors for bone formation and fracture repair. Nat Commun. 2017;8:2043. 29230039 10.1038/s41467-017-02171-2 PMC5725597

[26] Shen B , Tasdogan A , Ubellacker JM , et al. A mechanosensitive peri‐arteriolar niche for osteogenesis and lymphopoiesis. Nature. 2021;591:438‐444. 33627868 10.1038/s41586-021-03298-5 PMC7979521

[27] Delgado‐Calle J , Bellido T . The osteocyte as a signaling cell. Physiol Rev. 2022;102:379‐410. 34337974 10.1152/physrev.00043.2020 PMC8858675

[28] Lefebvre V , Angelozzi M , Haseeb A . SOX9 in cartilage development and disease. Curr Opin Cell Biol. 2019;61:39‐47. 31382142 10.1016/j.ceb.2019.07.008 PMC6956855

[29] Haseeb A , Kc R , Angelozzi M , et al. SOX9 keeps growth plates and articular cartilage healthy by inhibiting chondrocyte dedifferentiation/osteoblastic redifferentiation. Proc Natl Acad Sci USA. 2021;118:118. 10.1073/pnas.2019152118 PMC7923381 33597301

[30] Kim P , Park J , Lee DJ , et al. Mast4 determines the cell fate of MSCs for bone and cartilage development. Nat Commun. 2022;13:3960. 35803931 10.1038/s41467-022-31697-3 PMC9270402

[31] Huynh N , Zhang B , Guilak F . High‐depth transcriptomic profiling reveals the temporal gene signature of human mesenchymal stem cells during chondrogenesis. FASEB J. 2019;33:358‐372. 29985644 10.1096/fj.201800534R PMC6355072

[32] Baryawno N , Przybylski D , Kowalczyk MS , et al. A cellular taxonomy of the bone marrow stroma in homeostasis and leukemia. Cell. 2019;177:1915‐1932. 31130381 10.1016/j.cell.2019.04.040 PMC6570562

[33] Mizuhashi K , Ono W , Matsushita Y , et al. Resting zone of the growth plate houses a unique class of skeletal stem cells. Nature. 2018;563:254‐258. 30401834 10.1038/s41586-018-0662-5 PMC6251707

[34] Zuscik MJ , Hilton MJ , Zhang X , Chen D , O'Keefe RJ . Regulation of chondrogenesis and chondrocyte differentiation by stress. J Clin Invest. 2008;118:429‐438. 18246193 10.1172/JCI34174 PMC2214711

[35] Farquharson C , Jefferies D . Chondrocytes and longitudinal bone growth: the development of tibial dyschondroplasia. Poult Sci. 2000;79:994‐1004. 10901201 10.1093/ps/79.7.994

[36] Zhang P , Li X , Pan C , et al. Single‐cell RNA sequencing to track novel perspectives in HSC heterogeneity. Stem Cell Res Ther. 2022;13:39. 35093185 10.1186/s13287-022-02718-1 PMC8800338

[37] Seita J , Weissman IL . Hematopoietic stem cell: self‐renewal versus differentiation. WIREs Syst Biol Med. 2010;2:640‐653. 10.1002/wsbm.86 PMC2950323 20890962

[38] Boyce BF . Advances in the regulation of osteoclasts and osteoclast functions. J Dent Res. 2013;92:860‐867. 23906603 10.1177/0022034513500306 PMC3775372

[39] Yang H , Wang L , Shigley C , Yang W . Protein tyrosine phosphatases in skeletal development and diseases. Bone Res. 2022;10:10. 35091552 10.1038/s41413-021-00181-x PMC8799702

[40] Kong YY , Yoshida H , Sarosi I , et al. OPGL is a key regulator of osteoclastogenesis, lymphocyte development and lymph‐node organogenesis. Nature. 1999;397:315‐323. 9950424 10.1038/16852

[41] Li J , Sarosi I , Yan XQ , et al. RANK is the intrinsic hematopoietic cell surface receptor that controls osteoclastogenesis and regulation of bone mass and calcium metabolism. Proc Natl Acad Sci USA. 2000;97:1566‐1571. 10677500 10.1073/pnas.97.4.1566 PMC26475

[42] Bucay N , Sarosi I , Dunstan CR , et al. Osteoprotegerin‐deficient mice develop early onset osteoporosis and arterial calcification. Genes Dev. 1998;12:1260‐1268. 9573043 10.1101/gad.12.9.1260 PMC316769

[43] Simonet WS , Lacey DL , Dunstan CR , et al. Osteoprotegerin: a novel secreted protein involved in the regulation of bone density. Cell. 1997;89:309‐319. 9108485 10.1016/s0092-8674(00)80209-3

[44] McDonald MM , Khoo WH , Ng PY , et al. Osteoclasts recycle via osteomorphs during RANKL‐stimulated bone resorption. Cell. 2021;184:1940. 33798441 10.1016/j.cell.2021.03.010 PMC8024244

[45] Gao M , Liu X , Guo P , et al. Deciphering postnatal limb development at single‐cell resolution. Iscience. 2023;26:105808. 36619982 10.1016/j.isci.2022.105808 PMC9813795

[46] Tsukasaki M , Huynh NC , Okamoto K , et al. Stepwise cell fate decision pathways during osteoclastogenesis at single‐cell resolution. Nat Metab. 2020;2:1382‐1390. 33288951 10.1038/s42255-020-00318-y

[47] Hu K , Shang Z , Yang X , Zhang Y , Cao L . Macrophage polarization and the regulation of bone immunity in bone homeostasis. J Inflamm Res. 2023;16:3563‐3580. 37636272 10.2147/JIR.S423819 PMC10460180

[48] Chen S , Ni S , Liu C , et al. Neglected immunoregulation: M2 polarization of macrophages triggered by low‐dose irradiation plays an important role in bone regeneration. J Cell Mol Med. 2023;27:1095‐1109. 36929666 10.1111/jcmm.17721 PMC10098298

[49] Zha L , He L , Liang Y , et al. TNF‐α contributes to postmenopausal osteoporosis by synergistically promoting RANKL‐induced osteoclast formation. Biomed Pharmacother. 2018;102:369‐374. 29571022 10.1016/j.biopha.2018.03.080

[50] Bartok B , Firestein GS . Fibroblast‐like synoviocytes: key effector cells in rheumatoid arthritis. Immunol Rev. 2010;233:233‐255. 20193003 10.1111/j.0105-2896.2009.00859.x PMC2913689

[51] Culemann S , Grüneboom A , Nicolás‐Ávila JÁ , et al. Locally renewing resident synovial macrophages provide a protective barrier for the joint. Nature. 2019;572:670‐675. 31391580 10.1038/s41586-019-1471-1 PMC6805223

[52] Fahy N , de Vries‐van MM , Lehmann J , et al. Human osteoarthritic synovium impacts chondrogenic differentiation of mesenchymal stem cells via macrophage polarisation state. Osteoarthr Cartil. 2014;22:1167‐1175. 10.1016/j.joca.2014.05.021 24911520

[53] Zhang H , Lin C , Zeng C , et al. Synovial macrophage M1 polarisation exacerbates experimental osteoarthritis partially through R‐spondin‐2. Ann Rheum Dis. 2018;77:1524‐1534. 29991473 10.1136/annrheumdis-2018-213450

[54] Nygaard G , Firestein GS . Restoring synovial homeostasis in rheumatoid arthritis by targeting fibroblast‐like synoviocytes. Nat Rev Rheumatol. 2020;16:316‐333. 32393826 10.1038/s41584-020-0413-5 PMC7987137

[55] Wu Z , Ma D , Yang H , et al. Fibroblast‐like synoviocytes in rheumatoid arthritis: surface markers and phenotypes. Int Immunopharmacol. 2021;93:107392. 33529910 10.1016/j.intimp.2021.107392

[56] Han D , Fang Y , Tan X , et al. The emerging role of fibroblast‐like synoviocytes‐mediated synovitis in osteoarthritis: an update. J Cell Mol Med. 2020;24:9518‐9532. 32686306 10.1111/jcmm.15669 PMC7520283

[57] Bao C , Zhu S , Song K , He C . HK2: a potential regulator of osteoarthritis via glycolytic and non‐glycolytic pathways. Cell Commun Signal. 2022;20:132. 36042519 10.1186/s12964-022-00943-y PMC9426234

[58] Cao X , Wu S , Wang X , Huang J , Zhang W , Liang C . Receptor tyrosine kinase C‐kit promotes a destructive phenotype of FLS in osteoarthritis via intracellular EMT signaling. Mol Med. 2023;29:38. 36959556 10.1186/s10020-023-00633-6 PMC10037859

[59] Chen X , Gong W , Shao X , et al. METTL3‐mediated m(6)A modification of ATG7 regulates autophagy‐GATA4 axis to promote cellular senescence and osteoarthritis progression. Ann Rheum Dis. 2022;81:87‐99. 34706873 10.1136/annrheumdis-2021-221091

[60] Endisha H , Datta P , Sharma A , et al. MicroRNA‐34a‐5p promotes joint destruction during osteoarthritis. Arthritis Rheumatol. 2021;73:426‐439. 33034147 10.1002/art.41552 PMC7986901

[61] Mayor R , Theveneau E . The neural crest. Development. 2013;140:2247‐2251. 23674598 10.1242/dev.091751

[62] Soldatov R , Kaucka M , Kastriti ME , et al. Spatiotemporal structure of cell fate decisions in murine neural crest. Science. 2019;364:eaas9536. 31171666 10.1126/science.aas9536

[63] Le Douarin NM , Creuzet S , Couly G , Dupin E . Neural crest cell plasticity and its limits. Development. 2004;131:4637‐4650. 15358668 10.1242/dev.01350

[64] Liao J , Huang Y , Wang Q , et al. Gene regulatory network from cranial neural crest cells to osteoblast differentiation and calvarial bone development. Cell Mol Life Sci. 2022;79:158. 35220463 10.1007/s00018-022-04208-2 PMC11072871

[65] Kamalakar A , McKinney JM , Salinas DD , et al. JAGGED1 stimulates cranial neural crest cell osteoblast commitment pathways and bone regeneration independent of canonical NOTCH signaling. Bone. 2021;143:115657. 32980561 10.1016/j.bone.2020.115657 PMC9035226

[66] Simões‐Costa M , Bronner ME . Establishing neural crest identity: a gene regulatory recipe. Development. 2015;142:242‐257. 25564621 10.1242/dev.105445 PMC4302844

[67] Rocha M , Singh N , Ahsan K , Beiriger A , Prince VE . Neural crest development: insights from the zebrafish. Dev Dyn. 2020;249:88‐111. 31591788 10.1002/dvdy.122 PMC7273345

[68] Mori‐Akiyama Y , Akiyama H , Rowitch DH , de Crombrugghe B . Sox9 is required for determination of the chondrogenic cell lineage in the cranial neural crest. Proc Natl Acad Sci USA. 2003;100:9360‐9365. 12878728 10.1073/pnas.1631288100 PMC170923

[69] Fu M , Hu Y , Lan T , Guan KL , Luo T , Luo M . The Hippo signalling pathway and its implications in human health and diseases. Signal Transduct Tar. 2022;7:376. 10.1038/s41392-022-01191-9 PMC9643504 36347846

[70] Xu T , Wang W , Zhang S , Stewart RA , Yu W . Identifying tumor suppressors in genetic mosaics: the Drosophila lats gene encodes a putative protein kinase. Development. 1995;121:1053‐1063. 7743921 10.1242/dev.121.4.1053

[71] Cunningham R , Hansen CG . The Hippo pathway in cancer: YAP/TAZ and TEAD as therapeutic targets in cancer. Clin Sci. 2022;136:197‐222. 10.1042/CS20201474 PMC8819670 35119068

[72] Russell JO , Camargo FD . Hippo signalling in the liver: role in development, regeneration and disease. Nat Rev Gastroenterol Hepatol. 2022;19:297‐312. 35064256 10.1038/s41575-021-00571-w PMC9199961

[73] Wu S , Liu Y , Zheng Y , Dong J , Pan D . The TEAD/TEF family protein scalloped mediates transcriptional output of the Hippo growth‐regulatory pathway. Dev Cell. 2008;14:388‐398. 18258486 10.1016/j.devcel.2008.01.007

[74] Lorthongpanich C , Thumanu K , Tangkiettrakul K , et al. YAP as a key regulator of adipo‐osteogenic differentiation in human MSCs. Stem Cell Res Ther. 2019;10:402. 31852542 10.1186/s13287-019-1494-4 PMC6921580

[75] Pan JX , Xiong L , Zhao K , et al. YAP promotes osteogenesis and suppresses adipogenic differentiation by regulating β‐catenin signaling. Bone Res. 2018;6:18. 29872550 10.1038/s41413-018-0018-7 PMC5984632

[76] Hong JH , Hwang ES , McManus MT , et al. TAZ, a transcriptional modulator of mesenchymal stem cell differentiation. Science. 2005;309:1074‐1078. 16099986 10.1126/science.1110955

[77] Tang Y , Rowe RG , Botvinick EL , et al. MT1‐MMP‐dependent control of skeletal stem cell commitment via a β1‐integrin/YAP/TAZ signaling axis. Dev Cell. 2013;25:402‐416. 23685250 10.1016/j.devcel.2013.04.011 PMC3736823

[78] Li J , Yan JF , Wan QQ , et al. Matrix stiffening by self‐mineralizable guided bone regeneration. Acta Biomater. 2021;125:112‐125. 33582360 10.1016/j.actbio.2021.02.012

[79] Seo E , Basu‐Roy U , Gunaratne PH , et al. SOX2 regulates YAP1 to maintain stemness and determine cell fate in the osteo‐adipo lineage. Cell Rep. 2013;3:2075‐2087. 23791527 10.1016/j.celrep.2013.05.029 PMC5053763

[80] Kegelman CD , Nijsure MP , Moharrer Y , et al. YAP and TAZ promote periosteal osteoblast precursor expansion and differentiation for fracture repair. J Bone Miner Res. 2021;36:143‐157. 32835424 10.1002/jbmr.4166 PMC7988482

[81] Xiong J , Almeida M , O'Brien CA . The YAP/TAZ transcriptional co‐activators have opposing effects at different stages of osteoblast differentiation. Bone. 2018;112:1‐9. 29626544 10.1016/j.bone.2018.04.001 PMC5970058

[82] Suo J , Feng X , Li J , et al. VGLL4 promotes osteoblast differentiation by antagonizing TEADs‐inhibited Runx2 transcription. Sci Adv. 2020;6:6. 10.1126/sciadv.aba4147 PMC7608831 33097532

[83] Kegelman CD , Coulombe JC , Jordan KM , et al. YAP and TAZ mediate osteocyte perilacunar/canalicular remodeling. J Bone Miner Res. 2020;35:196‐210. 31610061 10.1002/jbmr.3876 PMC7066596

[84] Li W , Deng Y , Feng B , Mak KKL . Mst1/2 kinases modulate glucose uptake for osteoblast differentiation and bone formation. J Bone Miner Res. 2018;33:1183‐1195. 29474739 10.1002/jbmr.3413

[85] Zhang Q , Wu B , Yuan Y , et al. CGRP‐modulated M2 macrophages regulate osteogenesis of MC3T3‐E1 via Yap1. Arch Biochem Biophys. 2021;697:108697. 33232717 10.1016/j.abb.2020.108697

[86] Li Y , Yang S , Qin L , Yang S . TAZ is required for chondrogenesis and skeletal development. Cell Discov. 2021;7:26. 33879790 10.1038/s41421-021-00254-5 PMC8058044

[87] Deng Y , Wu A , Li P , et al. Yap1 regulates multiple steps of chondrocyte differentiation during skeletal development and bone repair. Cell Rep. 2016;14:2224‐2237. 26923596 10.1016/j.celrep.2016.02.021

[88] Goto H , Nishio M , To Y , et al. Loss of Mob1a/b in mice results in chondrodysplasia due to YAP1/TAZ‐TEAD‐dependent repression of SOX9. Development. 2018;145:dev159244. 29511023 10.1242/dev.159244

[89] Vanyai HK , Prin F , Guillermin O , et al. Control of skeletal morphogenesis by the Hippo‐YAP/TAZ pathway. Development. 2020;147:dev187187. 32994166 10.1242/dev.187187 PMC7673359

[90] Li M , Ning J , Wang J , Yan Q , Zhao K , Jia X . SETD7 regulates chondrocyte differentiation and glycolysis via the Hippo signaling pathway and HIF‐1α. Int J Mol Med. 2021;48:210. 34617577 10.3892/ijmm.2021.5043 PMC8510680

[91] Lundin V , Sugden WW , Theodore LN , et al. YAP regulates hematopoietic stem cell formation in response to the biomechanical forces of blood flow. Dev Cell. 2020;52:446‐460. 32032546 10.1016/j.devcel.2020.01.006 PMC7398148

[92] Althoff MJ , Nayak RC , Hegde S , et al. Yap1‐Scribble polarization is required for hematopoietic stem cell division and fate. Blood. 2020;136:1824‐1836. 32483624 10.1182/blood.2019004113 PMC7568035

[93] Lee DH , Kim TS , Lee D , Lim DS . Mammalian sterile 20 kinase 1 and 2 are important regulators of hematopoietic stem cells in stress condition. Sci Rep. 2018;8:942. 29343826 10.1038/s41598-018-19637-y PMC5772645

[94] Lee J , Youn BU , Kim K , et al. Mst2 controls bone homeostasis by regulating osteoclast and osteoblast differentiation. J Bone Miner Res. 2015;30:1597‐1607. 25761670 10.1002/jbmr.2503

[95] Yang W , Lu X , Zhang T , et al. TAZ inhibits osteoclastogenesis by attenuating TAK1/NF‐κB signaling. Bone Res. 2021;9:33. 34253712 10.1038/s41413-021-00151-3 PMC8275679

[96] Li H , Hu S , Wu R , et al. 11β‐hydroxysteroid dehydrogenase type 1 facilitates osteoporosis by turning on osteoclastogenesis through Hippo signaling. Int J Biol Sci. 2023;19:3628‐3639. 37496992 10.7150/ijbs.82933 PMC10367550

[97] Zhao L , Guan H , Song C , et al. YAP1 is essential for osteoclastogenesis through a TEADs‐dependent mechanism. Bone. 2018;110:177‐186. 29432919 10.1016/j.bone.2018.01.035

[98] Li Q , Han G , Liu D , Zhou Y . Force‐induced decline of TEA domain family member 1 contributes to osteoclastogenesis via regulation of osteoprotegerin. Arch Oral Biol. 2019;100:23‐32. 30771694 10.1016/j.archoralbio.2019.01.020

[99] Feng Y , Liang Y , Zhu X , et al. The signaling protein Wnt5a promotes TGFβ1‐mediated macrophage polarization and kidney fibrosis by inducing the transcriptional regulators Yap/Taz. J Biol Chem. 2018;293:19290‐19302. 30333225 10.1074/jbc.RA118.005457 PMC6302175

[100] Li C , Jin Y , Wei S , et al. Hippo signaling controls NLR family pyrin domain containing 3 activation and governs immunoregulation of mesenchymal stem cells in mouse liver injury. Hepatology. 2019;70:1714‐1731. 31063235 10.1002/hep.30700 PMC6819196

[101] Zhou X , Li W , Wang S , et al. YAP aggravates inflammatory bowel disease by regulating M1/M2 macrophage polarization and gut microbial homeostasis. Cell Rep. 2019;27:1176‐1189. 31018132 10.1016/j.celrep.2019.03.028

[102] Meli VS , Atcha H , Veerasubramanian PK , et al. YAP‐mediated mechanotransduction tunes the macrophage inflammatory response. Sci Adv. 2020;6:eabb8471. 33277245 10.1126/sciadv.abb8471 PMC7717914

[103] Yang J , Li S , Li Z , et al. Targeting YAP1‐regulated glycolysis in fibroblast‐like synoviocytes impairs macrophage infiltration to ameliorate diabetic osteoarthritis progression. Adv Sci. 2024;11:e2304617. 10.1002/advs.202304617 PMC10837355 38044289

[104] Bottini A , Wu DJ , Ai R , et al. PTPN14 phosphatase and YAP promote TGFβ signalling in rheumatoid synoviocytes. Ann Rheum Dis. 2019;78:600‐609. 30808624 10.1136/annrheumdis-2018-213799 PMC7039277

[105] Symons RA , Colella F , Collins FL , et al. Targeting the IL‐6‐Yap‐Snail signalling axis in synovial fibroblasts ameliorates inflammatory arthritis. Ann Rheum Dis. 2022;81:214‐224. 34844926 10.1136/annrheumdis-2021-220875 PMC8762018

[106] Zhou W , Shen Q , Wang H , et al. Knockdown of YAP/TAZ inhibits the migration and invasion of fibroblast synovial cells in rheumatoid arthritis by regulating autophagy. J Immunol Res. 2020;2020:9510594. 33145365 10.1155/2020/9510594 PMC7599417

[107] Du Y , Cui R , Tian N , Chen M , Zhang X‐L , Dai S‐M . Regulation of type I interferon signature by VGLL3 in the fibroblast‐like synoviocytes of rheumatoid arthritis patients via targeting the Hippo pathway. Arthritis Res Ther. 2022;24:188. 35941675 10.1186/s13075-022-02880-0 PMC9358906

[108] Collins FL , Roelofs AJ , Symons RA , et al. Taxonomy of fibroblasts and progenitors in the synovial joint at single‐cell resolution. Ann Rheum Dis. 2023;82:428‐437. 36414376 10.1136/ard-2021-221682 PMC9933170

[109] Hindley CJ , Condurat AL , Menon V , et al. The Hippo pathway member YAP enhances human neural crest cell fate and migration. Sci Rep. 2016;6:23208. 26980066 10.1038/srep23208 PMC4793290

[110] Wang J , Xiao Y , Hsu CW , et al. Yap and Taz play a crucial role in neural crest‐derived craniofacial development. Development. 2016;143:504‐515. 26718006 10.1242/dev.126920 PMC4760309

[111] Sun Z , Da FC , Moreno M , et al. FoxO6 regulates Hippo signaling and growth of the craniofacial complex. PLoS Genet. 2018;14:e1007675. 30286078 10.1371/journal.pgen.1007675 PMC6197693

[112] Zhao X , Tang L , Le TP , et al. Yap and Taz promote osteogenesis and prevent chondrogenesis in neural crest cells in vitro and in vivo. Sci Signal. 2022;15:eabn9009. 36282910 10.1126/scisignal.abn9009 PMC9938793

[113] Liu K , Ni JD , Li WZ , et al. The Sp1/FOXC1/HOTTIP/LATS2/YAP/β‐catenin cascade promotes malignant and metastatic progression of osteosarcoma. Mol Oncol. 2020;14:2678‐2695. 32634265 10.1002/1878-0261.12760 PMC7530777

[114] Li Y , Yang S , Yang S . Rb1 negatively regulates bone formation and remodeling through inhibiting transcriptional regulation of YAP in Glut1 and OPG expression and glucose metabolism in male mice. Mol Metab. 2022;66:101630. 36343919 10.1016/j.molmet.2022.101630 PMC9672361

[115] Larsson J , Ohishi M , Garrison B , et al. Nf2/merlin regulates hematopoietic stem cell behavior by altering microenvironmental architecture. Cell Stem Cell. 2008;3:221‐227. 18682243 10.1016/j.stem.2008.06.005 PMC4197168

[116] Martínez TI , Steimle JD , Zhao X , Wang J , Martin JF . LATS1/2 control TGFB‐directed epithelial‐to‐mesenchymal transition in the murine dorsal cranial neuroepithelium through YAP regulation. Development. 2022;149:dev200860. 36125128 10.1242/dev.200860 PMC9587805

[117] Goodwin AF , Chen CP , Vo NT , Bush JO , Klein OD . YAP/TAZ regulate elevation and bone formation of the mouse secondary palate. J Dent Res. 2020;99:1387‐1396. 32623954 10.1177/0022034520935372 PMC7580170

[118] Manderfield LJ , Engleka KA , Aghajanian H , et al. Pax3 and hippo signaling coordinate melanocyte gene expression in neural crest. Cell Rep. 2014;9:1885‐1895. 25466249 10.1016/j.celrep.2014.10.061 PMC4267159

[119] Zhang X , Cai D , Zhou F , et al. Targeting downstream subcellular YAP activity as a function of matrix stiffness with Verteporfin‐encapsulated chitosan microsphere attenuates osteoarthritis. Biomaterials. 2020;232:119724. 31918221 10.1016/j.biomaterials.2019.119724

[120] Deng Y , Lu J , Li W , et al. Reciprocal inhibition of YAP/TAZ and NF‐κB regulates osteoarthritic cartilage degradation. Nat Commun. 2018;9:4564. 30385786 10.1038/s41467-018-07022-2 PMC6212432

[121] Clevers H , Nusse R . Wnt/β‐catenin signaling and disease. Cell. 2012;149:1192‐1205. 22682243 10.1016/j.cell.2012.05.012

[122] Baron R , Kneissel M . WNT signaling in bone homeostasis and disease: from human mutations to treatments. Nat Med. 2013;19:179‐192. 23389618 10.1038/nm.3074

[123] Akoumianakis I , Polkinghorne M , Antoniades C . Non‐canonical WNT signalling in cardiovascular disease: mechanisms and therapeutic implications. Nat Rev Cardiol. 2022;19:783‐797. 35697779 10.1038/s41569-022-00718-5 PMC9191761

[124] Zhou T , Gao B , Fan Y , et al. Piezo1/2 mediate mechanotransduction essential for bone formation through concerted activation of NFAT‐YAP1‐ß‐catenin. Elife. 2020;9:9. 10.7554/eLife.52779 PMC7112954 32186512

[125] Azzolin L , Zanconato F , Bresolin S , et al. Role of TAZ as mediator of Wnt signaling. Cell. 2012;151:1443‐1456. 23245942 10.1016/j.cell.2012.11.027

[126] Jiao S , Li C , Hao Q , et al. VGLL4 targets a TCF4‐TEAD4 complex to coregulate Wnt and Hippo signalling in colorectal cancer. Nat Commun. 2017;8:14058. 28051067 10.1038/ncomms14058 PMC5216127

[127] Wang M , Zhao X , Qiu R , et al. Lymph node metastasis‐derived gastric cancer cells educate bone marrow‐derived mesenchymal stem cells via YAP signaling activation by exosomal Wnt5a. Oncogene. 2021;40:2296‐2308. 33654199 10.1038/s41388-021-01722-8 PMC7994201

[128] Wang P , Huang L , Yang F , Chen W , Bai D , Guo Y . YAP/TEAD1 and β‐catenin/LEF1 synergistically induce estrogen receptor α to promote osteogenic differentiation of bone marrow stromal cells. Medcomm. 2023;4:e246. 37197086 10.1002/mco2.246 PMC10183651

[129] Byun MR , Hwang JH , Kim AR , et al. Canonical Wnt signalling activates TAZ through PP1A during osteogenic differentiation. Cell Death Differ. 2014;21:854‐863. 24510127 10.1038/cdd.2014.8 PMC4013522

[130] Haffner‐Luntzer M , Ragipoglu D , Ahmad M , et al. Wnt1 boosts fracture healing by enhancing bone formation in the fracture callus. J Bone Miner Res. 2023;38:749‐764. 36891752 10.1002/jbmr.4797

[131] Yang B , Sun H , Song F , Yu M , Wu Y , Wang J . YAP1 negatively regulates chondrocyte differentiation partly by activating the β‐catenin signaling pathway. Int J Biochem Cell Biol. 2017;87:104‐113. 28438716 10.1016/j.biocel.2017.04.007

[132] Zhang Y , Zuo T , McVicar A , Yang HL , Li YP , Chen W . Runx1 is a key regulator of articular cartilage homeostasis by orchestrating YAP, TGFβ, and Wnt signaling in articular cartilage formation and osteoarthritis. Bone Res. 2022;10:63. 36307389 10.1038/s41413-022-00231-y PMC9616925

[133] Kumar D , Nitzan E , Kalcheim C . YAP promotes neural crest emigration through interactions with BMP and Wnt activities. Cell Commun Signal. 2019;17:69. 31228951 10.1186/s12964-019-0383-x PMC6589182

[134] Chen G , Deng C , Li YP . TGF‐β and BMP signaling in osteoblast differentiation and bone formation. Int J Biol Sci. 2012;8:272‐288. 22298955 10.7150/ijbs.2929 PMC3269610

[135] Hosokawa R , Urata M , Han J , et al. TGF‐beta mediated Msx2 expression controls occipital somites‐derived caudal region of skull development. Dev Biol. 2007;310:140‐153. 17727833 10.1016/j.ydbio.2007.07.038 PMC3337706

[136] Qiu T , Wu X , Zhang F , Clemens TL , Wan M , Cao X . TGF‐beta type II receptor phosphorylates PTH receptor to integrate bone remodelling signalling. Nat Cell Biol. 2010;12:224‐234. 20139972 10.1038/ncb2022 PMC3704184

[137] Luo K . Signaling cross talk between TGF‐β/Smad and other signaling pathways. Cold Spring Harb Perspect Biol. 2017;9(1):a022137. 27836834 10.1101/cshperspect.a022137 PMC5204325

[138] Wei Q , Holle A , Li J , et al. BMP‐2 signaling and mechanotransduction synergize to drive osteogenic differentiation via YAP/TAZ. Adv Sci. 2020;7:1902931. 10.1002/advs.201902931 PMC7404154 32775147

[139] Yin X , Choudhury M , Kang JH , et al. Hexokinase 2 couples glycolysis with the profibrotic actions of TGF‐β. Sci Signal. 2019;12:eaax4067. 31848318 10.1126/scisignal.aax4067

[140] de Araújo FV , Carrillo‐Gálvez AB , Martín F , Anderson P . TGF‐β and mesenchymal stromal cells in regenerative medicine, autoimmunity and cancer. Cytokine Growth Factor Rev. 2018;43:25‐37. 29954665 10.1016/j.cytogfr.2018.06.002

[141] Reichenbach M , Mendez PL , Da SMC , et al. Differential impact of fluid shear stress and YAP/TAZ on BMP/TGF‐β induced osteogenic target genes. Adv Biol. 2021;5:e2000051. 10.1002/adbi.202000051 36073990

[142] Yang B , Sun H , Xu X , Zhong H , Wu Y , Wang J . YAP1 inhibits the induction of TNF‐α‐stimulated bone‐resorbing mediators by suppressing the NF‐κB signaling pathway in MC3T3‐E1 cells. J Cell Physiol. 2020;235:4698‐4708. 31642068 10.1002/jcp.29348

[143] Wang L , You X , Lotinun S , Zhang L , Wu N , Zou W . Mechanical sensing protein PIEZO1 regulates bone homeostasis via osteoblast‐osteoclast crosstalk. Nat Commun. 2020;11:282. 31941964 10.1038/s41467-019-14146-6 PMC6962448

[144] Ding B , Xiao L , Xu H . YAP1 controls degeneration of human cartilage chondrocytes in response to mechanical tension. Cell Biol Int. 2022;46:1637‐1648. 35819082 10.1002/cbin.11851

[145] Yu H , Wang H , Liu J , Huang T , Man Y , Xiang L . The effect of ROS‐YAP crosstalk on osteoimmune response orchestrating osteogenesis. Cell Cycle. 2023;22:1391‐1405. 37161399 10.1080/15384101.2023.2211830 PMC10228400

[146] Dupont S , Morsut L , Aragona M , et al. Role of YAP/TAZ in mechanotransduction. Nature. 2011;474:179‐183. 21654799 10.1038/nature10137

[147] Aragona M , Panciera T , Manfrin A , et al. A mechanical checkpoint controls multicellular growth through YAP/TAZ regulation by actin‐processing factors. Cell. 2013;154:1047‐1059. 23954413 10.1016/j.cell.2013.07.042

[148] Wang L , You X , Zhang L , Zhang C , Zou W . Mechanical regulation of bone remodeling. Bone Res. 2022;10:16. 35181672 10.1038/s41413-022-00190-4 PMC8857305

[149] Shi H , Zhou K , Wang M , et al. Integrating physicomechanical and biological strategies for BTE: biomaterials‐induced osteogenic differentiation of MSCs. Theranostics. 2023;13:3245‐3275. 37351163 10.7150/thno.84759 PMC10283054

[150] Kim J , Jo H , Hong H , et al. Actin remodelling factors control ciliogenesis by regulating YAP/TAZ activity and vesicle trafficking. Nat Commun. 2015;6:6781. 25849865 10.1038/ncomms7781

[151] Halder G , Dupont S , Piccolo S . Transduction of mechanical and cytoskeletal cues by YAP and TAZ. Nat Rev Mol Cell Biol. 2012;13:591‐600. 22895435 10.1038/nrm3416

[152] Yin F , Yu J , Zheng Y , Chen Q , Zhang N , Pan D . Spatial organization of Hippo signaling at the plasma membrane mediated by the tumor suppressor Merlin/NF2. Cell. 2013;154:1342‐1355. 24012335 10.1016/j.cell.2013.08.025 PMC3835333

[153] Li W , Zhao J , Wang J , et al. ROCK‐TAZ signaling axis regulates mechanical tension‐induced osteogenic differentiation of rat cranial sagittal suture mesenchymal stem cells. J Cell Physiol. 2020;235:5972‐5984. 31970784 10.1002/jcp.29522

[154] Stanley A , Heo SJ , Mauck RL , Mourkioti F , Shore EM . Elevated BMP and mechanical signaling through YAP1/RhoA poises FOP mesenchymal progenitors for osteogenesis. J Bone Miner Res. 2019;34:1894‐1909. 31107558 10.1002/jbmr.3760 PMC7209824

[155] Elbediwy A , Vincent‐Mistiaen ZI , Spencer‐Dene B , et al. Integrin signalling regulates YAP and TAZ to control skin homeostasis. Development. 2016;143:1674‐1687. 26989177 10.1242/dev.133728 PMC4874484

[156] Wang S , Englund E , Kjellman P , et al. CCM3 is a gatekeeper in focal adhesions regulating mechanotransduction and YAP/TAZ signalling. Nat Cell Biol. 2021;23:758‐770. 34226698 10.1038/s41556-021-00702-0

[157] Chang L , Azzolin L , Di Biagio D , et al. The SWI/SNF complex is a mechanoregulated inhibitor of YAP and TAZ. Nature. 2018;563:265‐269. 30401838 10.1038/s41586-018-0658-1 PMC7612964

[158] Takeichi M . The cadherin superfamily in neuronal connections and interactions. Nat Rev Neurosci. 2007;8:11‐20. 17133224 10.1038/nrn2043

[159] Bertocchi C , Wang Y , Ravasio A , et al. Nanoscale architecture of cadherin‐based cell adhesions. Nat Cell Biol. 2017;19:28‐37. 27992406 10.1038/ncb3456 PMC5421576

[160] Benham‐Pyle BW , Pruitt BL , Nelson WJ . Cell adhesion. Mechanical strain induces E‐cadherin‐dependent Yap1 and β‐catenin activation to drive cell cycle entry. Science. 2015;348:1024‐1027. 26023140 10.1126/science.aaa4559 PMC4572847

[161] Giampietro C , Disanza A , Bravi L , et al. The actin‐binding protein EPS8 binds VE‐cadherin and modulates YAP localization and signaling. J Cell Biol. 2015;211:1177‐1192. 26668327 10.1083/jcb.201501089 PMC4687874

[162] Wu J , Minikes AM , Gao M , et al. Intercellular interaction dictates cancer cell ferroptosis via NF2‐YAP signalling. Nature. 2019;572:402‐406. 31341276 10.1038/s41586-019-1426-6 PMC6697195

[163] Ke W , Liao Z , Liang H , et al. Stiff substrate induces nucleus pulposus cell ferroptosis via YAP and N‐cadherin mediated mechanotransduction. Adv Healthc Mater. 2023;12:e2300458. 37022980 10.1002/adhm.202300458

[164] Zhang Z , Sha B , Zhao L , et al. Programmable integrin and N‐cadherin adhesive interactions modulate mechanosensing of mesenchymal stem cells by cofilin phosphorylation. Nat Commun. 2022;13:6854. 36369425 10.1038/s41467-022-34424-0 PMC9652405

[165] Zhang C , Zhu H , Ren X , et al. Mechanics‐driven nuclear localization of YAP can be reversed by N‐cadherin ligation in mesenchymal stem cells. Nat Commun. 2021;12:6229. 34711824 10.1038/s41467-021-26454-x PMC8553821

[166] Yu FX , Zhao B , Panupinthu N , et al. Regulation of the Hippo‐YAP pathway by G‐protein‐coupled receptor signaling. Cell. 2012;150:780‐791. 22863277 10.1016/j.cell.2012.06.037 PMC3433174

[167] Khalafalla FG , Greene S , Khan H , et al. P2Y(2) nucleotide receptor prompts human cardiac progenitor cell activation by modulating Hippo signaling. Circ Res. 2017;121:1224‐1236. 28923792 10.1161/CIRCRESAHA.117.310812 PMC5726767

[168] Mederos YSM , Storch U , Meibers S , et al. Gq‐coupled receptors as mechanosensors mediating myogenic vasoconstriction. EMBO J. 2008;27:3092‐3103. 18987636 10.1038/emboj.2008.233 PMC2599876

[169] Gao J , He L , Zhou L , et al. Mechanical force regulation of YAP by F‐actin and GPCR revealed by super‐resolution imaging. Nanoscale. 2020;12:2703‐2714. 31950964 10.1039/c9nr09452k

[170] Cai H , Xu Y . The role of LPA and YAP signaling in long‐term migration of human ovarian cancer cells. Cell Commun Signal. 2013;11:31. 23618389 10.1186/1478-811X-11-31 PMC3655373

[171] Yu FX , Zhang Y , Park HW , et al. Protein kinase A activates the Hippo pathway to modulate cell proliferation and differentiation. Genes Dev. 2013;27:1223‐1232. 23752589 10.1101/gad.219402.113 PMC3690396

[172] Zhong G , Su S , Li J , et al. Activation of Piezo1 promotes osteogenic differentiation of aortic valve interstitial cell through YAP‐dependent glutaminolysis. Sci Adv. 2023;9:eadg478. 10.1126/sciadv.adg0478 PMC10413650 37267365

[173] Ying K , Liu H , Tarkhov AE , et al. Causality‐enriched epigenetic age uncouples damage and adaptation. Nat Aging. 2024;4:231‐246. 38243142 10.1038/s43587-023-00557-0 PMC11070280

[174] Bao H , Cao J , Chen M , et al. Biomarkers of aging. Sci China Life Sci. 2023;66:893‐1066. 37076725 10.1007/s11427-023-2305-0 PMC10115486

[175] Sladitschek‐Martens HL , Guarnieri A , Brumana G , et al. YAP/TAZ activity in stromal cells prevents ageing by controlling cGAS‐STING. Nature. 2022;607:790‐798. 35768505 10.1038/s41586-022-04924-6 PMC7613988

[176] Xu X , Shen X , Wang J , et al. YAP prevents premature senescence of astrocytes and cognitive decline of Alzheimer's disease through regulating CDK6 signaling. Aging Cell. 2021;20:e13465. 34415667 10.1111/acel.13465 PMC8441453

[177] Yue X , Cui J , Sun Z , et al. Nuclear softening mediated by Sun2 suppression delays mechanical stress‐induced cellular senescence. Cell Death Dis. 2023;9:167. 10.1038/s41420-023-01467-1 PMC10192198 37198162

[178] Du K , Maeso‐Díaz R , Oh SH , et al. Targeting YAP‐mediated HSC death susceptibility and senescence for treatment of liver fibrosis. Hepatology. 2023;77:1998‐2015. 36815382 10.1097/HEP.0000000000000326 PMC10416614

[179] Yu B , Huo L , Liu Y , et al. PGC‐1α controls skeletal stem Cell fate and bone‐fat balance in osteoporosis and skeletal aging by inducing TAZ. Cell Stem Cell. 2018;23:193‐209. 30017591 10.1016/j.stem.2018.06.009 PMC6322535

[180] Santinon G , Brian I , Pocaterra A , et al. dNTP metabolism links mechanical cues and YAP/TAZ to cell growth and oncogene‐induced senescence. EMBO J. 2018;37:e97780. 29650681 10.15252/embj.201797780 PMC5983219

[181] Ren X , Hu B , Song M , et al. Maintenance of nucleolar homeostasis by CBX4 alleviates senescence and osteoarthritis. Cell Rep. 2019;26:3643‐3656. 30917318 10.1016/j.celrep.2019.02.088

[182] Chen F , Hou W , Yu X , et al. CBX4 deletion promotes tumorigenesis under Kras(G12D) background by inducing genomic instability. Signal Transduct Target Ther. 2023;8:343. 37696812 10.1038/s41392-023-01623-0 PMC10495400

[183] Li CJ , Xiao Y , Yang M , et al. Long noncoding RNA Bmncr regulates mesenchymal stem cell fate during skeletal aging. J Clin Invest. 2018;128:5251‐5266. 30352426 10.1172/JCI99044 PMC6264619

[184] Zhang L , Zhang C , Zheng J , et al. miR‐155‐5p/Bmal1 modulates the senescence and osteogenic differentiation of mouse BMSCs through the Hippo signaling pathway. Stem Cell Rev Rep. 2024;20:554‐567. 38150082 10.1007/s12015-023-10666-3 PMC10837250

[185] Driskill JH , Pan D . Control of stem cell renewal and fate by YAP and TAZ. Nat Rev Mol Cell Biol. 2023;24:895‐911. 37626124 10.1038/s41580-023-00644-5

